# Self-assembled low-molecular-weight gelator injectable microgel beads for delivery of bioactive agents†

**DOI:** 10.1039/d0sc06296k

**Published:** 2021-02-02

**Authors:** Carmen C. Piras, Alasdair G. Kay, Paul G. Genever, David K. Smith

**Affiliations:** Department of Chemistry, University of York Heslington York YO10 5DD UK david.smith@york.ac.uk; Department of Biology, University of York Heslington York YO10 5DD UK

## Abstract

We report the preparation of hybrid self-assembled microgel beads by combining the low molecular weight gelator (LMWG) DBS-CONHNH_2_ and the natural polysaccharide calcium alginate polymer gelator (PG). Microgel formulations based on LMWGs are extremely rare due to the fragility of the self-assembled networks and the difficulty of retaining any imposed shape. Our hybrid beads contain interpenetrated LMWG and PG networks, and are obtained by an emulsion method, allowing the preparation of spherical gel particles of controllable sizes with diameters in the mm or μm range. Microgels based on LMWG/alginate can be easily prepared with reproducible diameters <1 μm (*ca.* 800 nm). They are stable in water at room temperature for many months, and survive injection through a syringe. The rapid assembly of the LMWG on cooling plays an active role in helping control the diameter of the microgel beads. These LMWG microbeads retained the ability of the parent gel to deliver the bioactive molecule heparin, and in cell culture medium this enhanced the growth of human mesenchymal stem cells. Such microgels may therefore have future applications in tissue repair. This approach to fabricating LMWG microgels is a platform technology, which could potentially be applied to a variety of different functional LMWGs, and hence has wide-ranging potential.

## Abstract 2

We report microgel beads with diameters of *ca.* 800 nm based on interpenetrating networks of a low-molecular-weight gelator and a polymer gelator, and demonstrate their use as heparin delivery vehicles to enhance stem cell growth.

## Graphical abstract

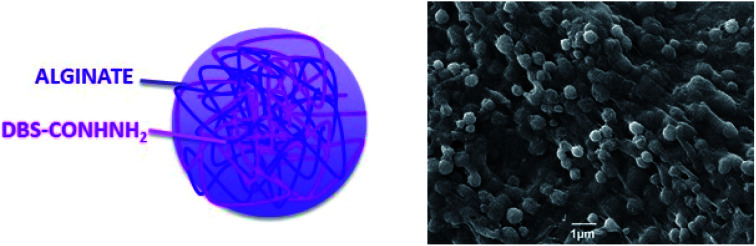

## Introduction

In the last few years, nanogels and microgels have been the focus of growing attention for their applications in biomedicine and drug delivery.^1^ These are small spherical gel particles with diameters in the nanometre or micrometre range respectively. Formed by colloidal networks, they display the features of hydrogels (*i.e.* water retention and capability to trap molecules), whilst having the advantages of their small dimensions, such as higher surface area, greater exchange rates and faster responses to environmental changes.^2^ These properties make such materials highly valuable carriers for the delivery of desired cargos (*e.g.* active pharmaceutical ingredients, biological agents, or stem cells).

Hydrogels can be obtained from polymer gelators (PGs) or small molecules (low molecular weight gelators – LMWGs).^3^ Due to the robustness and ease of manipulation of polymers, most nano- and microgels reported in the literature are based on PGs.^4^ Conversely, micro/nano-gel systems based on LMWGs are exceptionally rare. In terms of microparticle assembly, a poor solvent was employed by Hudalla and co-workers to encourage microgel formation from self-assembling peptides, giving microscale objects with diameters of 5–12.5 μm.^5^ Ulijn and co-workers combined a microfluidic flow system with biocatalysis to generate an LMWG *in situ* and form gel microparticles (30–50 μm) from water-in-oil microdroplets.^6^ Others have gone on to further elaborate LMWG assembly in water droplets using microfluidic systems.^7^ There have also been reports of LMWG microshells in which self-assembly is mediated at the interface of an oil-in-water emulsion.^8^ AT the sub-micron level, in 2017, Miravet and co-workers reported the generation of nanosized spherical objects by injecting an LMWG dissolved in a good solvent into a poor solvent, and suggested the objects obtained were particles formed in the initial stages of the nucleation of self-assembly.^9^ Maintaining the stability of these LMWG nanoparticles over time was challenging as a result of their tendency to aggregate. By using gelatin, the team were able to mediate the aggregation of these objects into larger assemblies, thus obtaining ill-defined ‘sheaf-like’ micro-particles. Miravet and co-workers also used sonication on solvated xerogels to generate similar nanoparticulate materials.^10^ Most recently, they formed nanoparticles (*ca.* 50 nm) from LMWGs within a stabilising liposome shell that was subsequently removed.^11^ These nanoparticle LMWGs are very small, and it is difficult to fully understand their internal structuring and stability.

The combination of a LMWG with a PG to form a hybrid gel is a known strategy to enhance the mechanical properties and stability of a stimulus-responsive self-assembled gel, potentially providing spatial control over the gelation event – a key target of LMWG research.^12^ In recent work, we reported macroscale self-assembled gel beads, with diameters of *ca.* 3.0–3.6 mm, formed by the PG calcium alginate and the LMWG 1,3:2,4-di-(4-acylhydrazide)-benzylidenesorbitol (DBS-CONHNH_2_).^13^ Using temporal and thermal control of the gelation process, we were able to achieve spatial control of the two gel networks within these beads, with the PG forming the shell of the bead, which was then filled with self-assembled LMWG.

**Fig. 1:**
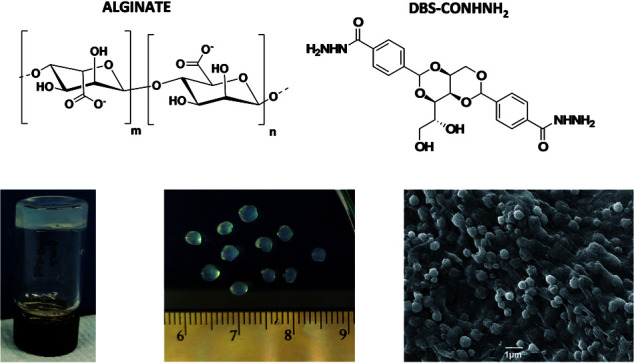
Chemical structures of sodium alginate and DBS-CONHNH_2_ and, from left to right, images of DBS-CONHNH_2_/alginate gels in vial, millimetre scale beads and microgel beads prepared by the emulsion method.

Given the clear need for simple, reproducible fabrication methods for LMWG-based microgels, we reasoned that, by modifying the preparation method, we could potentially achieve greater control of the size of the resulting gel beads. We therefore targeted ‘sizing down’ these hybrid gel beads from the millimetre length scale to the microscale, and in particular, to the reproducible formation of sub-micron-sized gel beads. This paper describes a fabrication technique which allows the formation of self-assembled supramolecular gel beads with well-defined diameters of *ca.* 800 nm, and subsequently stabilises them using calcium alginate. For comparison, equivalent gels are also made in vials and as millimetre-scale gel beads (Fig. 1). This constitutes a rare example of a stable LMWG-based microgel. The fabrication and stabilisation method is very simple and could easily be applied to other LMWGs, to give microgels with a wide range of chemical compositions, and potential applications.

## Results and discussion

**Fig. 2:**
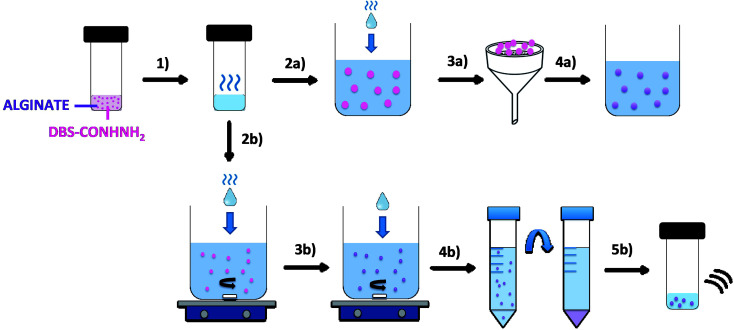
Schematic representation of DBS-CONHNH_2_/alginate gel beads and microgels preparation by the emulsion method. (1) A mixture of DBS-CONHNH_2_ (0.3% wt/vol) and alginate (0.5% wt/vol) is heated until complete dissolution of the LMWG. To obtain hybrid gel beads, the hot solution is added dropwise to paraffin oil (2a). The gel droplets are then collected by filtration (3a) and transferred to a CaCl_2_ solution (5.0% wt/vol) to cross-link the alginate (4a). Alternatively, to obtain microgels, the DBS-CONHNH_2_/alginate hot solution is added dropwise to a mixture of paraffin oil and Span80 under stirring (2b). After 1 h, CaCl_2_ (5.0% wt/vol) is added and the emulsion is stirred for another 20 min (3b). The sample is then transferred into falcon tubes and the microgel particles are purified through multiple washings with petroleum ether, ethanol and water and centrifugation cycles (4b). Finally, the sample is transferred into a sample vial and sonicated to help the dispersion of the particles (5b).

The LMWG DBS-CONHNH_2_ was synthesized by our previously-reported method.^14^ It forms thermally-responsive bio-compatible hydrogels *via* a heat-cool cycle, which have been explored for applications including drug delivery, tissue engineering and environmental remediation.^14,15^ The PG based on alginic acid is commercially available – the polymer forms ionically cross-linked hydrogels when in contact with bivalent cations (*e.g.* Ca^2+^).^16^ In our previous work,^13^ we generated core–shell beads by simply dripping a hot solution of LMWG and sodium alginate into a solution of calcium ions. The droplet size controlled the size of the beads that were formed (typically *ca.* 3 mm), and the rapid formation of calcium alginate at the periphery led to a core–shell morphology. To generate smaller gel beads based on DBS-CONHNH_2_/alginate hydrogels we targeted systems in which the gel networks were woven together rather than organised into core–shell structures, using an emulsion-based fabrication method to give more control over bead size (Fig. 2).

**Fig. 3:**
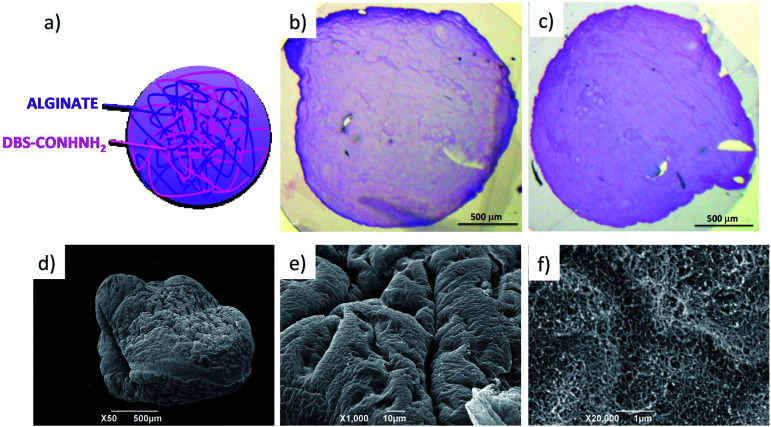
(a) Schematic representation of the spatial arrangement of the two gelators within the gel beads. (b and c) Optical microscopy of the cross-section of the gel beads embedded in resin and coloured using toluidine blue (scale bars 500 μm). (d) SEM of a whole gel bed, (e) gel bead surface and (f) cross-section (scale bars 500, 10 and 1 μm).

Initially, a hot aqueous DBS-CONHNH_2_/alginate mixture was added dropwise (20 μL drops) to paraffin oil and left undisturbed for 20 min to initially allow the formation of the DBS-CONHNH_2_ network on cooling (Fig. 2, step 2a). These LMWG gel beads were filtered off (Fig. 2, step 3a), then transferred to a CaCl_2_ bath (5.0% wt/vol) to slowly induce the formation of the second gel network (Fig. 2, step 4a). We reasoned this second slow step would allow diffusion of calcium ions through the pre-formed LMWG bead meaning the two gel networks would be woven throughout the gel beads, rather than organised into a core–shell structure as in our previous work. The initial gel beads fabricated using this approach had a diameter of 3.0–3.5 mm, as in our previous work, controlled by the drop size. Differently to our previous work, there is clear temporal control, with the LMWG hydrogel beads forming first in the water droplets suspended in the paraffin oil, and then the PG network being used to stabilise it in a second step. The diameter could be varied on the millimetre length-scale by changing the volume of the drops of hot aqueous solution added to the paraffin oil. Optical microscopy of the cross-section of the gel beads clearly showed a uniform texture, very different from the core–shell spatial arrangement we had previously observed in our DBS-CONHNH_2_/alginate gel beads,^13^ thus confirming that the two individual networks were woven within the beads (Fig. 3 and S19†). To obtain insight into the morphology of the gel bead surface and cross-section, we performed SEM microscopy of the gel bead surface and cross-section. The surface of our hybrid gel beads appeared to be wrinkled and densely packed, and the cross-section showed a nanofibrillar network, confirming that the incorporated gelators were present in their self-assembled state (Fig. 3) in the interior of the beads.

The amount of LMWG incorporated into each gel bead was calculated by ^1^H NMR. Five gel beads were isolated and dried under vacuum. The resulting solid beads were added to DMSO-d_6_, which only dissolved the DBS-CONHNH_2_, but not the alginate (Fig. S1†). The amount of LMWG was calculated by comparison of the integrals of the aromatic protons to that of an internal standard (CH_3_CN). In principle, 50 gel beads (20 μL volume each) could be prepared from 1 mL of water containing DBS-CONHNH_2_ (0.3% wt/vol, 6.32 μmol) and sodium alginate (0.5% wt/vol). If the DBS-CONHNH_2_ was fully incorporated into the gel beads and evenly distributed, each bead should contain *ca.* 0.12 μmol of LMWG. The NMR study indicated *ca.* 0.11 μmol of LMWG in each bead. This experiment was highly reproducible and we are therefore confident that >90% of the LMWG is incorporated within these mm-scale LMWG/PG gel beads.

Once we had demonstrated the efficiency of this two-step fabrication method, we wanted to scale-down the size of the resulting gel beads from mm-scale to μm-scale. To achieve this, we applied rapid stirring to break up larger water droplets and added a stabilising surfactant to help homogenise the system. We therefore added the DBS-CONHNH_2_/alginate hot aqueous solution drop-wise (20 μL drops) to a mixture of paraffin oil and the surfactant Span80 under stirring (Fig. 2, step 2b). The surfactant was selected to help the dispersion of the particles in the emulsion and reduce aggregation. This gives rise, in the first step, to self-assembled LMWG microgel beads as the hot solution cools in the paraffin oil. The mixture was stirred for 1 h, and CaCl_2_ (5.0% wt/vol) was then added dropwise (20 μL drops) to the emulsion, which was stirred for another 20 min (Fig. 2, step 3b). This second step assembles the calcium alginate PG, which will act as a stabilising network for the initially formed LMWG microbeads. The resulting microgel particles were isolated by centrifugation (Fig. 2, step 4b) and washed multiple times with petroleum ether, ethanol and water to ensure complete removal of the paraffin oil. Finally, the sample is sonicated to disperse the beads (Fig. 2, step 5b). In this approach, the calcium alginate PG is not being used to help form the gel microbeads, but rather just to stabilise them.

Alginate-only microgels were also prepared using the same method applied for the hybrid microgels described above, with the only difference being that, since alginate is water-soluble and does not require a heat-cool cycle to form hydrogels, we did not heat the sample prior to addition to the paraffin oil/Span80 mixture.

**Fig. 4:**
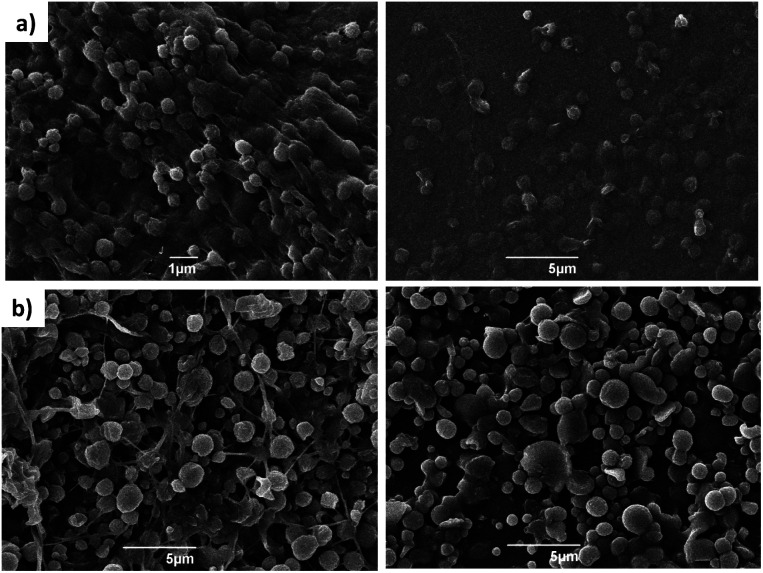
SEM images of (a) DBS-CONHNH_2_/alginate two-component microgels and (b) alginate microgels. The images on the left are freshly prepared and those on the right are after 30 days. Scale bars: 1 μm ((a) left) and 5 μm ((a) right and (b) left and right).

**Fig. 5:**
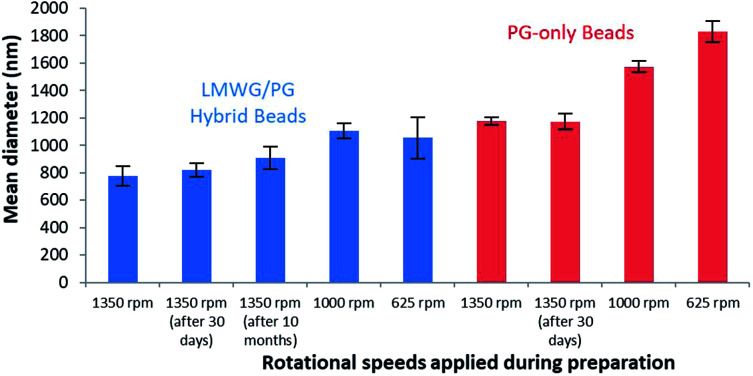
Size distribution by volume measured by DLS of DBS-CONHNH_2_/alginate two-component microgels (blue) and alginate-only microgels (red) prepared using different rotational speeds.

The formation of spherical gel beads with diameters in the μm (and indeed sub-μm) range was confirmed by scanning electron microscopy (SEM, Fig. 4, S20 and S21†) and dynamic light scattering (Fig. S9 and S17†). The diameters of the DBS-CONHNH_2_/alginate microgels measured from the SEM images were 0.4–0.8 μm, with the majority in the range of 0.4–0.6 μm. The alginate-only system had slightly larger gel bead diameters, with the majority being above 1.0 μm. DLS indicated that the size distribution of the isolated microgels was 615–955 nm for the DBS-CONHNH_2_/alginate two-component system and slightly larger at 955–1430 nm for the alginate-only microbeads (Fig. 5). The diameters measured using SEM were slightly smaller than those measured by DLS, probably as a result of dehydration during sample preparation for microscopy analysis.

To understand the difference in size between the two-component microgel beads and the slightly larger alginate-only beads we reflect in more detail on the fabrication method. The LMWG, DBS-CONHNH_2_, assembles in the first step on cooling, with the hybrid DBS-CONHNH_2_/alginate self-assembled microgel particles rapidly forming their LMWG network after dropwise addition of the hot LMWG/PG mixture into the room temperature paraffin. We hypothesize that, since the LMWG gel particles are ‘pre-formed’ in this way, they do not increase in size on stirring for 1 h prior to addition of the CaCl_2_ to crosslink the PG in the second stabilising step. In contrast, when the alginate-only emulsion is prepared, the alginate liquid droplets are stirred for 1 h before cross-linking and gel formation occurs on addition of CaCl_2_. During this time, we propose that the particle size could increase slightly due to droplet–droplet collision (coalescence) and fusion of the particles. This therefore demonstrates a positive effect of the presence of the LMWG in the formulation of these microgel beads – it can essentially act as a thermally-controlled ‘setting agent’ in the first step of the process, helping control the dimensions of the beads being generated prior to their stabilisation with calcium alginate in the second step. LMWG assembly has similarly been shown to have beneficial effects in creating curable inks for 3D-printing applications.^17^

We explored the effect of different stirring speeds on the size of the DBS-CONHNH_2_/alginate and alginate-only microgel beads. This study was performed using a 2 cm stirrer bar in an 80 mL volume beaker placed on a standard magnetic stirrer hot plate. DLS analysis of microgels prepared at different stirring speeds (*i.e.* 1350, 1000 and 650 rpm) showed that higher stirring speeds led to the formation of significantly smaller particles (Fig. 5, S9 and S12†). The mean diameter of the two-component microgel beads dropped from 1150 nm at 625 rpm to 785 nm at 1350 rpm. Since the best results in terms of size distribution were obtained using a rotational speed of 1350 rpm, with sub-micron-sized beads being reproducibly generated, we decided to apply this as standard for the preparation of microgel beads. Alginate-only beads showed a similar dependence of diameter on stirring rate (Fig. 5, S17 and S18†), albeit with the beads being consistently larger (see discussion above). It is worth noting that sub-micron-sized beads based on an LMWG, such as those obtained here, remain very rare. Further studies comparing the mean diameter of DBS-CONHNH_2_/alginate microgel beads prepared using different concentrations of alginate, CaCl_2_, Span80 and different oil/water ratios were also performed (Table S2, Fig. S9 and S13–S16†). Changing the oil/water ratio had limited impact on bead size. Overall, increasing the loading of Span80 from 2% to 4% led to larger beads. At lower levels of CaCl_2_, we observed undesirable bimodal distributions of particle diameters. At higher alginate loadings, the diameter of the beads increased somewhat, consistent with the view that as alginate begins to dominate the hybrid gel, the LMWG is less able to exert its control over particle diameter.

To verify the stability of the microgels over time, we re-analysed the DBS-CONHNH_2_/alginate and alginate microgel samples after 30 days of storage in water at room temperature (Fig. 5). SEM and DLS showed that the samples were stable over time (Fig. 4, S10, S17, S20 and S21†). A small size increase of the hybrid gel beads was observed from a mean diameter of 785 nm to 820 nm, although within error range, this may indicate slight particle aggregation. The hybrid gel beads were investigated again by DLS after standing for 10 months (pandemics have some advantages, Fig. S10†), and pleasingly, the bead diameter was still below 1 μm, being 910 nm. Overall, this indicates excellent long-term stability of the microgel beads in solution.

**Fig. 6:**
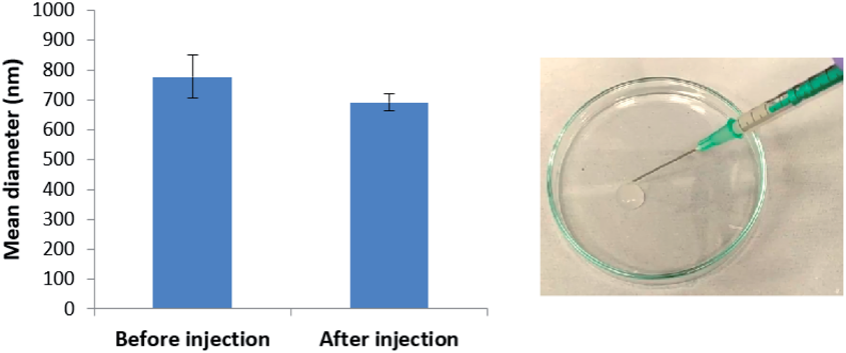
(Left) Mean diameter of DBS-CONHNH_2_/alginate two-component microgels prepared with mixing at 1350 rpm, before and after they have been injected through a syringe. (Right) Photograph of DBS-CONHNH_2_/alginate two-component microgels being injected through a syringe.

We tested the stability of our microgel particles to injection through a standard syringe needle. After injection through the needle, the bead sizes were determined by DLS (Fig. 6 and S11†). Before injection the average diameter was 775 nm, whereas after injection it was 690 nm. These values were almost within error of one another – the small difference may suggest that smaller microgel beads pass slightly more effectively through the syringe. Pleasingly the results indicate excellent stability towards syringe injection. Injectable microgels have potential for use in clinical applications such as drug delivery or tissue engineering (see discussion below).

The efficiency of the fabrication method of these hybrid microgels was then evaluated by ^1^H NMR. A microgel sample was prepared by the emulsion method described above from DBS-CONHNH_2_ (0.3% wt/vol, 6.32 μmol – in 1 mL of water) and alginate (0.5% wt/vol). The particles isolated after the washing and centrifugation steps, were dried under vacuum. The resulting solid was dissolved in DMSO-d_6_ and a known amount of CH_3_CN added as an internal standard (Fig. S2†). The sample analysed contained 3.04 μmol of DBS-CONHNH_2_, which corresponds to *ca.* 48% of the LMWG added. This experiment was repeated on different microgel batches and was reproducible. Clearly, more LMWG is lost during the preparation of microgel beads than during the formation of macroscale beads described above. Analysis prior to and after washing indicated that some of the LMWG is lost during the relatively extensive washing steps. Given the smaller diameters of the microgel beads, such losses might be expected to be more significant, as there is effectively a greater amount of bead surface exposed to the environment. Nonetheless, this experiment clearly demonstrates that the LMWG is present within the hybrid microgel beads.

**Fig. 7:**
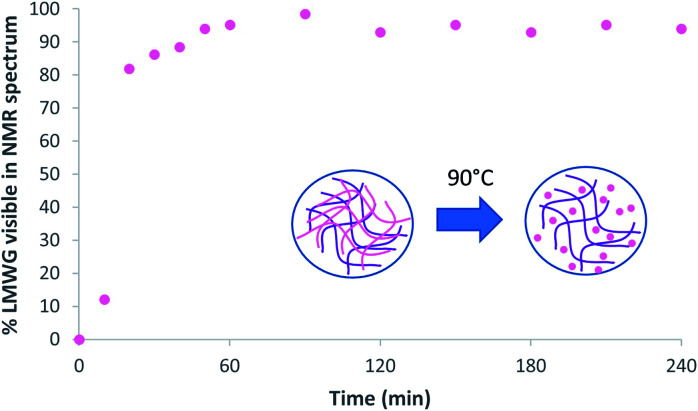
Percentage of DBS-CONHNH_2_ visualised by ^1^H NMR, and hence in the mobile liquid-like phase, after heating at 90 °C in the NMR spetrometer for different amounts of time. Schematic indicating thermally-induced disassembly of the LMWG network within a LMWG/PG hybrid gel microbead.

Solution phase ^1^H NMR spectroscopy was then used on a sample of the microgel beads in D_2_O. Within gels, ^1^H NMR is an excellent technique to determine whether an LWMG is in the assembled state, or whether it is mobile in the solution phase.^18^ If the gelator is in the assembled state, then no NMR signal is detected as a result of its low mobility, however, if it is mobile, then ^1^H NMR signals are observed. This allows quantification of the self-assembly of an LMWG within the gel. In this case, solution phase ^1^H NMR of the microgel particles gave no signal (Fig. S3†), indicating that the DBS-CONHNH_2_ within the hybrid gel beads is in the self-assembled state. Heating the NMR sample is then an effective way of demonstrating that the DBS-CONHNH_2_ is in self-assembled form (nanofibre disassembly is thermally triggered in this case). We therefore heated the gel beads to 90 °C and with the use of an internal standard, were able to determine the concentration of mobile DBS-CONHNH_2_ (Fig. S4 and Table S1†) This experiment demonstrated that within an hour, as expected, the DBS-CONHNH_2_ completely disassembled into a mobile ‘liquid-like’ state (Fig. 7). This therefore provides clear evidence that DBS-CONHNH_2_ is indeed self-assembled with the hybrid gel microbeads.

We also performed variable temperature UV-vis studies (Fig. S5 and S6†). These indicated that at room temperature, there was no leaching of DBS-CONHNH_2_ from the gel microbeads. However, on raising the temperature to 90 °C, a significant amount of the LMWG was released into the solution phase. The self-assembled DBS-CONHNH_2_ network undergoes thermally-induced disassembly, and the gelator becomes part of the mobile liquid-like phase, and hence able to diffuse out of the microbeads. Once again, therefore, this provides clear evidence that the LMWG is indeed self-assembled within the hybrid gel microbeads.

The hybrid microgel beads were also studied by IR spectroscopy (Fig. S7 and S8†). In the hybrid microgel sample, the alginate O–H band (3338 cm^−1^) shifted to 3326 cm^−1^ in the presence of DBS-CONHNH_2_, whereas the C=O band shifted from 1601 to 1591 cm^−1^. These data confirm the presence of self-assembled DBS-CONHNH_2_ within the gel microbeads and suggest a degree of non-covalent interaction between the two gel networks similar to those reported by us previously.^13^

To provide a preliminary demonstration of a possible use of these hybrid microgel beads we explored controlled release. We decided to focus on the natural polysaccharide heparin, which is an anti-coagulant drug and a potent modulator of growth factor receptor binding.^19^ This bioactive molecule is in clinical use as an anti-coagulant, and controlled release of this drug is relevant in the treatment of deep vein thrombosis in hospital settings.^20^ Furthermore, it is known to promote cell growth and proliferation and it is thus also relevant in tissue engineering and regenerative medicine.^21^ Studies on heparin release from bulk samples of DBS-CONHNH_2_ gels and hybrid gels based on this LMWG were previously reported by us and the system is quite well understood, making it an ideal candidate to benchmark the performance of these gel microbeads.^22^

We applied our already optimised protocol based on the use of the heparin binder Mallard Blue (MalB)^23^ and monitored the release of heparin from the different DBS-CONHNH_2_/alginate two-component gel formulations into 10 mM Tris–HCl/150 mM NaCl buffer (pH 7.4). Release at 37 °C was studied by analysing the absorbance at 615 nm by UV-vis spectroscopy at regular time intervals. All the gels, gel beads and microgels were initially loaded with heparin by soaking each sample in a concentrated heparin solution (2 mL, 1 mM). After 24 hours, the heparin solution was removed, and used to quantify the exact amount of heparin incorporated into each gel sample by UV-vis spectroscopy. To investigate heparin release, buffer (10 mM Tris–HCl/150 mM NaCl to 2 mL) was then placed on top of each gel and 65 μL aliquots were collected over time, added to MalB and analysed by UV. All experiments were performed in triplicate.

**Fig. 8:**
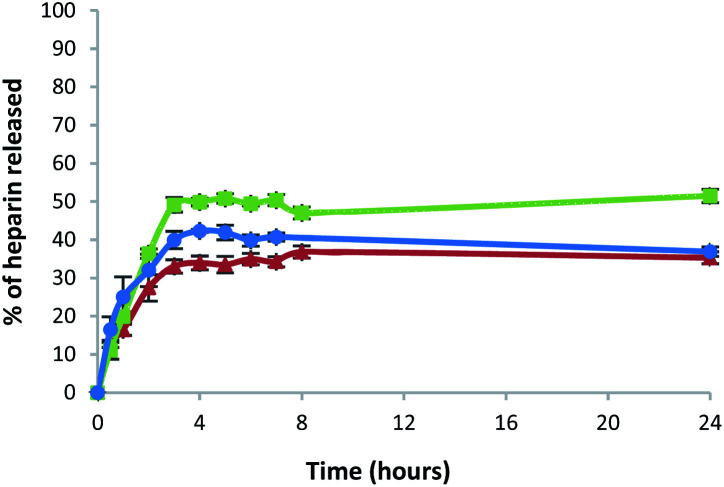
Percentage of heparin released over time in 10 mM Tris–HCl/150 mM NaCl buffer from: DBS-CONHNH_2_/alginate multicomponent gel in vial (red triangles), gel beads (green squares) and microgel beads (blue circles).

All the gels released heparin in broadly similar ways. Heparin release was slightly lower for the gel prepared in a sample vial, compared with the gel beads and microbeads (Fig. 8 and Table S3†). After 5 h, the percentage of heparin released into Tris–HCl buffer for the hybrid gel in a vial was *ca.* 33%, whereas *ca.* 50% was released from the gel beads and 41% from the gel microbeads. It is worth noting that heparin release is limited to *ca.* 50% because we did not exchange buffer during the experiment – this was a deliberate choice to simulate conditions in the cell growth studies (see below). Overall, however, the results are similar in each case, demonstrating that the heparin release function of these gels is effectively retained in the new microbead format.

Heparin is a biologically-relevant molecule, which can exhibit potent effects on cell proliferation, and is hence of some interest in tissue regeneration therapies.^24^ We therefore decided to verify if our gels could achieve controlled release of heparin in a cell culture environment, and hence influence cell growth.

**Fig. 9:**
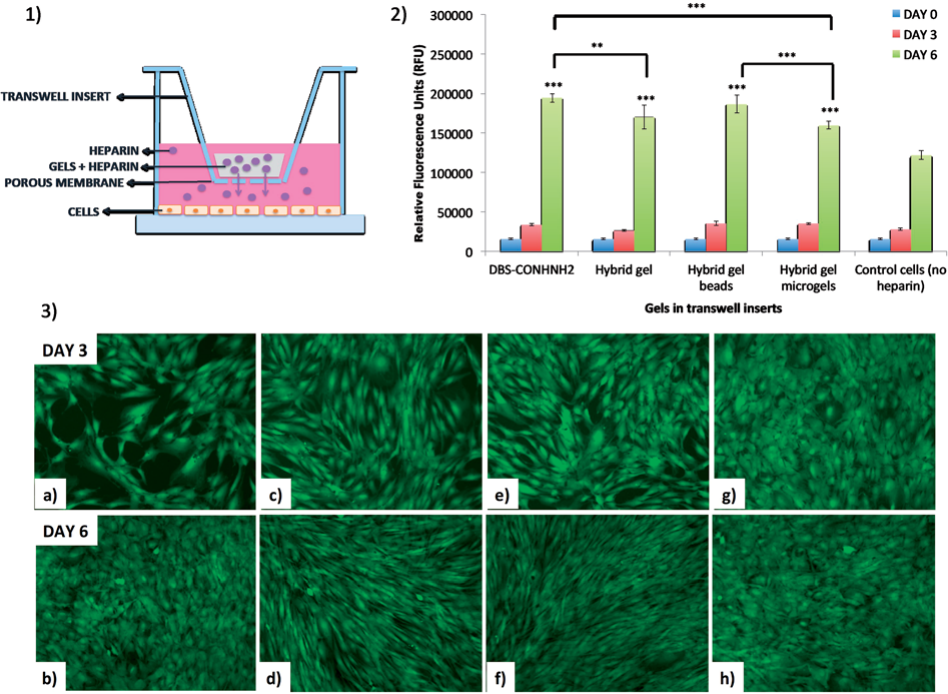
(1) Schematic representation of heparin loaded gels in transwells. (2) Deep blue metabolic activity assay results at day 0, 3 and 6 for the different gels loaded with 0.1 mg of heparin. Statistical significance (comparing viability at day 6 of the cells that received heparin from the different types of gels and the cells that were not exposed to heparin) is denoted by ** = *p* < 0.01 and *** = *p* < 0.001. (3) Fluorescence microscopy images at day 3 and 6 of calcein AM stained cells exposed to heparin (0.1 mg) released from DBS-CONHNH_2_ gels (respectively a and b), DBS-CONHNH_2_/alginate gels (respectively c and d), gel beads (respectively e and f) and microgels (respectively g and h).

This study was conducted using transwell inserts bearing a permeable membrane at the bottom (0.4 μm pores) (Fig. 9). The gels were directly prepared into the transwell inserts in a 75 μL volume (DBS-CONHNH_2_ and DBS-CONHNH_2_/alginate two-component gels) or placed into the inserts after preparation and sterilization. The different gel systems were loaded with equal amounts of heparin and the transwell inserts transferred to a 24-well plate in which Y201 immortalised human mesenchymal stem cells^25^ (25 000 cells per well) had been seeded 24 hours earlier and covered with Dulbecco's Modified Eagle's cell culture medium (DMEM). We reasoned the permeable membrane on the inserts would allow the heparin to diffuse and reach the cells, but prevent the diffusion of the microgel beads.

We first tested the stability of the gel microbeads in cell culture medium. Gel microbeads were dispersed in the medium and stored in the incubator at 37 °C for one week. After one week, the DMEM was removed by centrifugation and the sample washed multiple times with extra pure water. DLS was used to assess the size of the gel microbeads, which was 874.5 ± 60 nm (Fig. S11†). This is comparable to the diameters reported above (Fig. 5) and indicates good microgel stability in medium. The cells were stained with calcein and imaged by at day 0, 3 and 6. The fluorescence microscopy images show the cells were alive throughout the experiment (Fig. 9, S24 and S25†). An increase in the number of viable cells over time indicated the cells were proliferating. By day 6, the cells had achieved a high level of confluence, and therefore the experiment was stopped.

Cell metabolic activity was monitored prior to addition of the transwell inserts containing the heparin-loaded gels (day 0), and at days 3 and 6 using the Deep Blue Cell Viability kit (BioLegend), based on the reduction of resazurin (blue) into resorufin (pink) by the action of metabolic enzymes in live cells. The collected results showed a clear increase in fluorescence intensity over time for all cells. Importantly, the fluorescence signal produced by the cells that received heparin from the gels was significantly higher at day 6 than the signal produced by the control cells (Fig. 9 and S24†). This was true for all of the heparin-loaded gels, demonstrating that all formats of the hybrid gel are capable of heparin encapsulation and release. After 6 days, cell metabolic activity is *ca.* 50% greater for those cells grown in the presence of heparin-loaded gels. Furthermore, the results also suggest that no cytotoxic components are released from the gels. This is not surprising considering that both the individual components of our hybrid gels are known to be biocompatible.^15*c*,26^ These results therefore show that our hybrid DBS-CONHNH_2_/alginate gel, in different formats, including as injectable microgel beads, could be successfully used in a cell culture environment as a reservoir system for the controlled release of heparin.

## Conclusions

We have reported a water-in-oil emulsion-based method to obtain DBS-CONHNH_2_/alginate two-component self-assembled gel beads with interwoven supramolecular gel networks. This method is very simple and has the potential to be easily applied to other LMWGs. Importantly, we were able to ‘size-down’ the diameter of these gel beads to sub-μm size, to obtain DBS-CONHNH_2_/alginate hybrid microgels. This was achieved by vigorous stirring and the addition of a surfactant to stabilise the water droplets. Importantly, in step 1, this initially gives rise to microscale gel beads based solely on the LMWG, which are then stabilised in step 2, on exposure to CaCl_2_ by the formation of the calcium alginate PG network. The resulting microscale beads have diameters of *ca.* 800 nm and are a rare example of microgel particles based on a LMWG. The LMWG plays an active role in helping control the diameter of the gel microbeads, acting as a thermal setting agent that stabilises the water droplets during microbead preparation. The resulting beads were stable in water at room temperature for as long as 10 months. The microgels were stable to injection and also exhibited good stability in cell culture medium. It is noteworthy that we can reproducibly generate and then stabilise sub-micron-sized gel particles based on self-assembled LMWGs – such objects are very rare.

We loaded the bioactive molecule heparin into these hybrid gels and then tested its controlled release. Heparin release into buffer demonstrated that these hybrid gels could successfully encapsulate and release this biologically-relevant polysaccharide with this property being retained by the gel microbeads. We tested the ability of these hybrid heparin-loaded gels to release their cargo and influence the growth of human stem cells. Cell metabolic activity was significantly increased (by 50%) after 6 days in the presence of the heparin-loaded gels. We reason that the microgels in particular may be useful for *in vivo* use, where they could potentially be injected into damaged tissue and actively assist with tissue regrowth and recovery – indeed PG microgels are being widely explored for use in this setting.^1,27^

The collected results show the considerable potential of these hybrid DBS-CONHNH_2_/alginate gels. In addition to their biocompatible nature, the fact that they can be formulated into systems with different shapes, sizes and spatial arrangements, makes this gel system a versatile platform technology for a wide range of controlled release applications. It would be interesting to load other bioactive agents into these microgels – work in this regard is currently in progress. Importantly, the microgel fabrication and stabilisation technology described here should be broadly applicable to a wide range of LMWGs, and we believe it can potentially open up a variety of new applications for self-assembled gels.

## Conflicts of interest

There are no conflicts to declare.

## Supplementary Material

SC-012-D0SC06296K-s001
