# Supplementary material for: Self-assembled low-molecular-weight gelator injectable microgel beads for delivery of bioactive agents
Source: Chem Sci. 2021 Feb 2;12(11):3958–65. doi: 10.1039/d0sc06296k (PMC8179440; doi:10.1039/d0sc06296k)
Supplement: SC-012-D0SC06296K-s001 [file SC-012-D0SC06296K-s001.pdf]

Electronic Supplementary Material (ESI) for Chemical Science.
This journal is © The Royal Society of Chemistry 2021

Self-assembled low-molecular-weight gelator injectable microgel beads for delivery of bioactive agents

Carmen C. Piras,[a] Alasdair G. Kay,[b] Paul G. Genever [b] and David K. Smith *[a]

a: Department of Chemistry, University of York, Heslington, York, YO10 5DD, UK

david.smith@york.ac.uk

b: Department of Biology, University of York, Heslington, York, YO10 5DD, UK

SUPPORTING INFORMATION

S1 General experimental methods

S2 Gel preparation

S3 NMR studies

S4 Variable Temperature UV-Vis Spectroscopy

S5 Infrared (IR) spectroscopy

S6 Dynamic Light Scattering (DLS)

S7 Optical microscopy

S8 Scanning Electron Microscopy (SEM)

S9 Release studies

S10 Biological studies

S11 References

S1 General Experimental Methods

All compounds used in synthesis and analysis were purchased from standard commercial suppliers and used as received. The alginate employed in all the experiments was bought from Sigma Aldrich as sodium salt (2% viscosity). The Deep Blue cell viability kit was purchased from BioLegend and the calcein AM for live cell staining from Cambridge Bioscience. The synthesis of DBS-$CONHNH_2$ was performed in good yields applying previously reported methods.[1,2] ^1H NMR spectra were recorded using a Jeol 400 spectrometer (^1H 400 MHz). Samples were prepared in DMSO-d_6 and chemical shifts (δ) are reported in parts per million (ppm). IR spectra of xerogels were recorded on a PerkinElmer Spectrum Two FT-IR spectrometer. Dynamic Light Scattering Measurements were performed on a Malvern Zetasizer Nano. Optical microscopy images were obtained using a Zeiss Axiocam camera on a Zeiss stereo microscope. SEM images were taken using a JEOL JSM-7600F field emission SEM. Gel bead and microgel diameters were measured using the *ImageJ* software. UV-vis spectra were collected on a UV-2401 PC spectrophotometer. Fluorescence measurements for the cell viability assay were performed using a BMG Labtech Clariostar Plate Reader. Fluorescence microscopy images were obtained using a fluorescence microscope.

S2 Gel preparation

S2.1 DBS-$CONHNH_2$/alginate two-component gel beads by emulsion. DBS-$CONHNH_2$ (0.3% wt/vol in 1 mL final total volume) was suspended in water (0.5 mL) and sonicated to help the dispersion of the solid particles. An aqueous alginate solution (1.0% wt/vol - 0.5 mL) was subsequently added. The resulting suspension was heated until complete dissolution of the DBS-$CONHNH_2$. The hot solution was then added dropwise (20 μL/drop) to paraffin oil (40 mL). The droplets were left undisturbed for 20 mins to allow the formation of the DBS-$CONHNH_2$ network. The gel beads were then transferred to a $CaCl_2$ solution (5.0% wt/vol, 50 mL) and gently mixed for another 20 mins. After this time, to remove residual paraffin oil, the gel beads were immersed in petroleum ether (30 mL, 10 mins), then EtOH (30 mL, 10 mins) and water (30 mL, 10 mins). When necessary, the washings were performed multiple times. The gel beads were then stored in water.

S2.2 Alginate gel beads. Alginate gel beads were prepared by dropwise addition (20 μL/drop) of an aqueous alginate solution (0.8% wt/vol) to a $CaCl_2$ solution (5.0% wt/vol). The obtained beads were collected by filtration and washed with water multiple times.

S2.3 DBS-CONHNH$_2$/alginate two-component microgels by surfactant-stabilised emulsion. DBS-CONHNH$_2$ (0.3% wt/vol in 1 mL final total volume) was suspended in water (0.5 mL) and sonicated to help dispersion of the solid particles. An aqueous alginate solution (1.0% wt/vol - 0.5 mL) was subsequently added. The resulting suspension was heated until complete dissolution of DBS-CONHNH$_2$. The hot solution was added dropwise (20 μL/drop) to a mixture of paraffin oil (40 mL) and Span80 (2.0%, 0.8 mL) under stirring. The mixture was stirred for 1h. After 1h, $CaCl_2$ (5.0% wt/vol, 1 mL) was added dropwise to the emulsion (20 μL/drop), which was stirred for another 20 min. After this time, the emulsion was centrifuged for 5 mins and the supernatant removed. The resulting pellet was then dispersed in petroleum ether and centrifuged. This process was repeated two more times with petroleum ether, then ethanol (three centrifuge cycles) and finally water (three centrifuge cycles). After the last centrifuge cycle, the pellet was dispersed in extra pure water (4 mL), transferred to a vial and sonicated for 20 mins to help dispersion of the microgel particles.

S2.4 Alginate microgels. Alginate microgels were prepared by dropwise addition (20 μL/drop) of an aqueous alginate solution (0.8% wt/vol) to a mixture of paraffin oil (40 mL) and Span80 (2.0%, 0.8 mL) under stirring. The mixture as stirred for 1h. After 1h, $CaCl_2$ (5.0% wt/vol, 1 mL) was added dropwise to the emulsion (20 μL/drop), which was stirred for another 20 mins. After this time, the emulsion was centrifuged for 5 mins and the supernatant removed. The resulting pellet was then dispersed in petroleum ether and centrifuged again. This process was repeated two more times with petroleum ether, then ethanol (three centrifuge cycles) and finally water (three centrifuge cycles). After the last centrifuge cycle, the pellet was dispersed in extra pure water (4 mL), transferred in a vial and sonicated for 20 mins to help the dispersion of the microgel particles.

S2.5 DBS-CONHNH$_2$ gels in sample vials. DBS-CONHNH$_2$ (0.3% wt/vol) was suspended in water (1 mL). The suspension was sonicated to help the dispersion of the solid particles and then heated until complete dissolution of the compound. The sample was left undisturbed to cool, allowing gel formation in a few minutes.

S2.6 Alginate gels in sample vials. Alginate gels were prepared by adding a $CaCl_2$ solution (5.0% wt/vol – 1 mL) to an aqueous alginate solution (0.8% wt/vol) for 30 mins. Gelation occurred immediately. The excess of $CaCl_2$ solution was then removed and the gels were washed with water multiple times.

S2.7 DBS-CONHNH$_2$/alginate two-component gels in sample vials. DBS-CONHNH$_2$ (0.3% wt/vol in 1 mL final total volume) was suspended in water (0.5 mL) and sonicated to help dispersion of the solid particles. An aqueous alginate solution (1.0% wt/vol - 0.5 mL) was then added. The resulting suspension was heated until complete dissolution of the DBS-CONHNH$_2$. The sample was left for a few hours to allow the formation of the DBS-CONHNH$_2$ network. A solution of $CaCl_2$ (5.0 % wt/vol – 1 mL) was then added on top of each gel to crosslink the alginate chains for 30 min. The excess of $CaCl_2$ solution was then removed and the gels were washed with water multiple times.

S3 NMR studies

S3.1 DBS-CONHNH$_2$/alginate two-component gel beads by emulsion. ^{1}H NMR was employed to calculate the exact amount of DBS-CONHNH$_2$ incorporated into the DBS-CONHNH$_2$/alginate two-component gel beads prepared by emulsion. The gel beads used for this experiment were prepared by combining DBS-CONHNH$_2$ (0.3 % wt/vol) and alginate (0.5 % wt/vol) as described in Section S2.1. Five gel beads were isolated and dried under high vacuum. The resulting solid was dissolved in DMSO-d$_6$ (0.7 mL), and acetonitrile (3.0 μL) was added as an internal standard. To make sure that all the DBS-CONHNH$_2$ was dissolved, the sample was ground and then sonicated for 30 min. The ^{1}H NMR spectrum was recorded and the concentration of the LMWG calculated by comparison of the integrals of relevant peaks (DBS-CONHNH$_2$ aromatic peaks δ = 7.53 and 7.83 ppm) to that of acetonitrile (δ = 2.09 ppm). To ensure the results were reproducible, this experiment was performed on two different batches of gel beads. It is noted that due to the low solubility of alginate in DMSO-d$_6$, the alginate peaks were not visible.

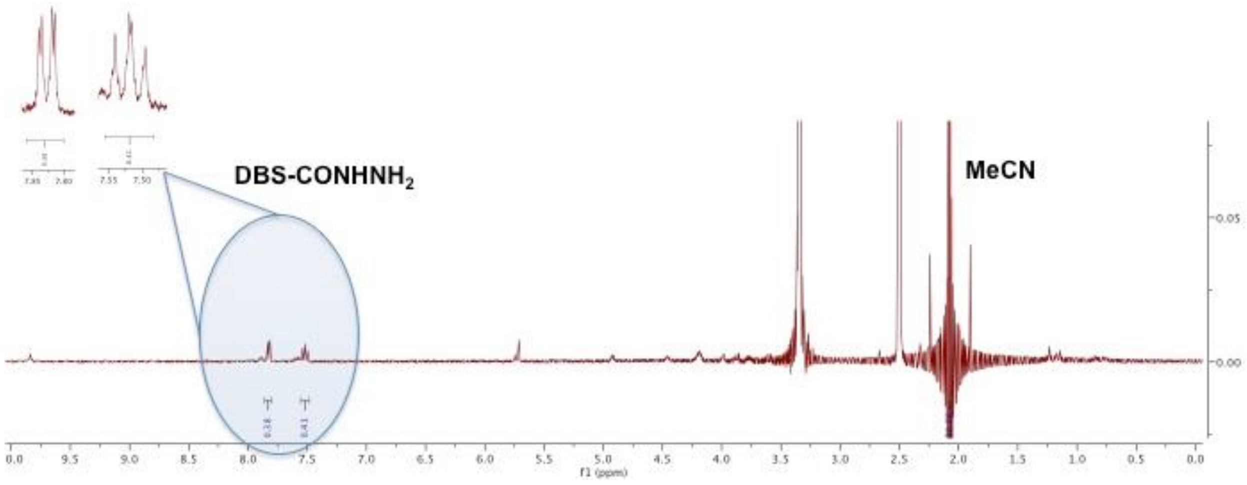

Figure S1. ^{1}H NMR of five dried DBS-CONHNH$_2$/alginate gel beads prepared by emulsion.

S3.2 DBS-CONHNH$_2$/alginate two-component microgels by surfactant-stabilised emulsion. ^{1}H NMR was employed to calculate the exact amount of DBS-CONHNH$_2$ incorporated into the DBS-CONHNH$_2$/alginate microgel particles and to estimate the yield of the preparation method. The microgels used for this experiment were prepared by combining DBS-CONHNH$_2$ (0.3 % wt/vol) and alginate (0.5 % wt/vol) as described in Section S2.3. The microgel samples were isolated and dried under high vacuum. The resulting solid was dissolved in DMSO-d$_6$ (0.7 mL), and acetonitrile (2.0 μL) was added as an internal standard. To make sure that all the DBS-CONHNH$_2$ was dissolved, the sample was sonicated for 30 min. The ^{1}H NMR spectrum was recorded and the concentration of the LMWG calculated by comparison of the integrals of relevant peaks (DBS-CONHNH$_2$ aromatic peaks δ = 7.53 and 7.83 ppm) to that of acetonitrile (δ = 2.09 ppm). To ensure the results were reproducible, this experiment was performed on two different batches of microgels. It is noted that due to the low solubility of alginate in DMSO-d$_6$, the alginate peaks were not visible.

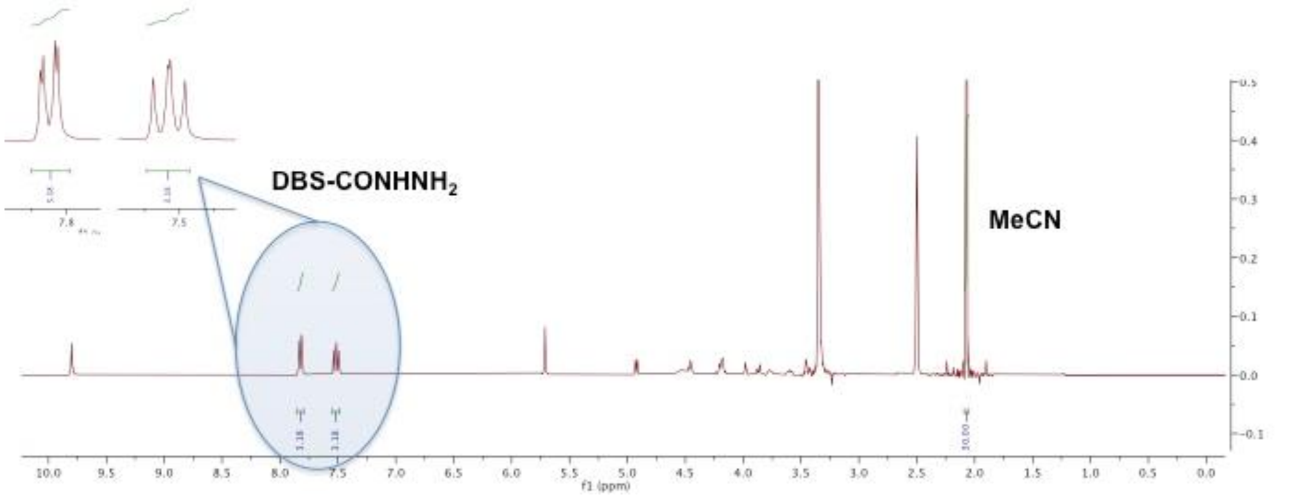

Figure S2. ^{1}H NMR of DBS-CONHNH$_2$/alginate dried microgels prepared by surfactant-stabilised emulsion.

S3.2. NMR verification of the self-assembled state of DBS-CONHNH$_2$ incorporated into the DBS-CONHNH$_2$/alginate microgel beads. The microgels used for this experiment were prepared by combining DBS-CONHNH$_2$ (0.3 % wt/vol) and alginate (0.5 % wt/vol) as described in Section S2.3, using D_2O instead of water. The microgel particles isolated were dispersed in D_2O (0.75 mL), transferred into a NMR tube and anhydrous MeCN (3.0 μL) was then added. The ^{1}H NMR spectrum was recorded and the amount of mobile components was calculated by comparison of the integrals of relevant peaks (DBS-CONHNH$_2$ aromatic peaks δ = 7.53 and 7.83 ppm) to that of

acetonitrile (δ = 2.09 ppm). The absence of peaks in the 1H NMR confirms that the two gelators incorporated into the microgel particles are in their self-assembled state.

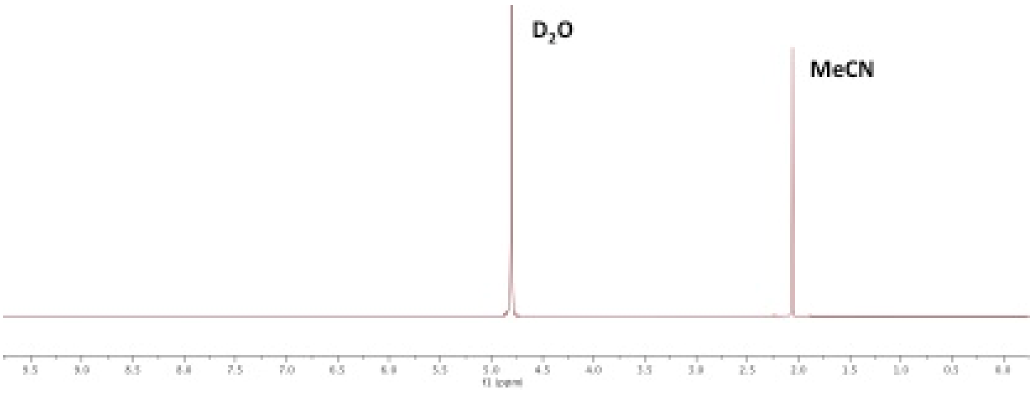

Figure S3. ^1H NMR of DBS-$CONHNH_2$/alginate microgels in D_2O.

S3.3 NMR study over time at 90°C. ^1H NMR was employed to monitor the disruption of the DBS-$CONHNH_2$ network incorporated into the DBS-$CONHNH_2$/alginate microgel particles over time. The microgels used for this experiment were prepared as follows. DBS-$CONHNH_2$ (0.3% wt/vol in 1 mL final total volume) was suspended in D_2O (0.5 mL) and sonicated to help the dispersion of the solid particles. An alginate solution in D_2O (1.0% wt/vol - 0.5 mL) was subsequently added. The resulting suspension was heated until complete dissolution of the DBS-$CONHNH_2$. The hot solution was then added dropwise (20 μL/drop) to paraffin oil (40 mL) under stirring. The mixture was stirred for 1h. After 1h, $CaCl_2$ (5.0% wt/vol, 1 mL in D_2O) was added dropwise to the emulsion (20 μL/drop), which was stirred for another 20 mins. After this time, the emulsion was centrifuged for 5 mins and the supernatant removed. The resulting pellet was then dispersed in D_2O and centrifuged again. The full purification process with organic solvents to remove residual paraffin oil was not performed to avoid the presence of excessive water in the ^1H NMR. After the last centrifugation step, a 100 μL aliquot of the resulting pellet was dispersed in 0.75 mL D_2O and transferred into a NMR tube. DMSO (2 μL) was added as an internal standard. The sample was then placed in the spectrometer. A ^1H NMR was immediately recorded to confirm that the DBS-$CONHNH_2$ incorporated into the microgel beads was in its self-assembled state. The sample was then heated to 90 °C. Spectra were recorded at 90 °C every 10 minutes for 60 minutes and then every 30 minutes for 12 hours. The concentration of the mobile components was calculated by comparison of the integrals of relevant peaks (DBS-$CONHNH_2$ aromatic peaks δ = 7.55 and 7.62) to that of DMSO (δ = 2.50 ppm). The obtained data are reported in Table S1.

The total amount of DBS-CONHNH$_2$ released from the microgels corresponds to 0.28 mg. Considering that a 100 μL aliquot was analysed (i.e. 10% of the 1 mL volume used to prepare the original sample), we calculated that 2.8 mg of DBS-CONHNH$_2$ were incorporated into the sample, which corresponds to c.a. 93% of the amount loaded (3.0 mg/mL). However, it is important to highlight that purification was not undertaken. The NMR experiment performed to evaluate the efficiency of the fabrication method showed that, when purification is performed, 48% of the initial amount of DBS-CONHNH$_2$ loaded is isolated. This overnight NMR experiment therefore, confirms that the rest of the LMWG is lost during the purification steps, which clearly affects the total amount of DBS-CONHNH$_2$/alginate microgel particles isolated.

Table S1. Percentage of unbound DBS-CONHNH$_2$ into DBS-CONHNH$_2$/alginate microgel beads (100 μL aliquot of the original sample) over time at a constant temperature of 90°C.

**Time (mins)**	**Unbound DBS-CONHNH$_2$ in 100 μL aliquot of microgel beads (mmoles)**	**% of unbound DBS-CONHNH$_2$ in 100 μL aliquot of microgel beads**	**Time (mins)**	**Unbound DBS-CONHNH$_2$ in 100 μL aliquot of microgel beads (mmoles)**	**% of unbound DBS-CONHNH$_2$ in 100 μL aliquot of microgel beads**
0	0.000077	0	360	0.000588	96.26
10	0.000518	12.17	390	0.000581	92.94
20	0.000546	81.87	420	0.000588	91.83
30	0.00056	86.30	450	0.000588	92.94
40	0.000595	88.51	480	0.000609	92.94
50	0.000602	94.04	510	0.000609	96.26
60	0.000623	95.15	540	0.000602	96.26
90	0.000588	98.47	570	0.000595	95.15
120	0.000602	92.94	600	0.000588	94.04
150	0.000588	95.15	630	0.000609	92.94
180	0.000602	92.94	660	0.000609	96.26
210	0.000595	95.15	690	0.000595	96.26
240	0.000609	94.04	720	0.000588	94.04
270	0.000602	96.26	750	0.000595	95.15
300	0.000595	95.15	780	0.000609	94.04
330	0.000609	94.04	810	0.000602	96.26

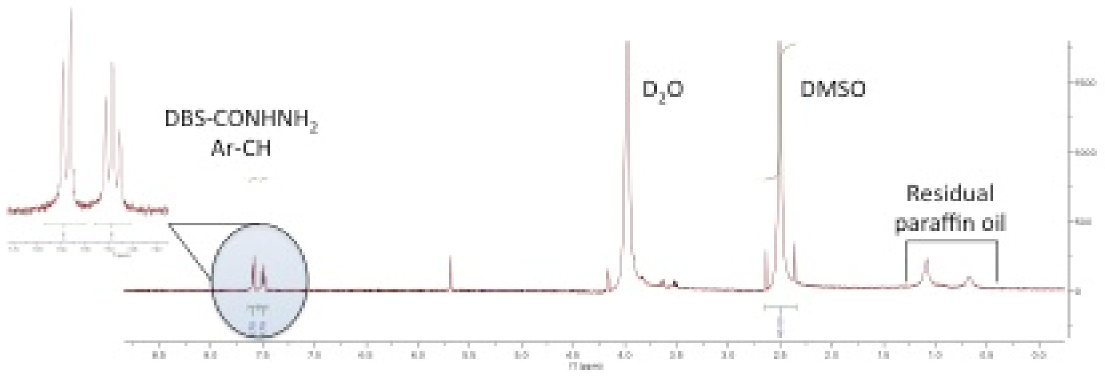

Figure S4. ^{1}H NMR of DBS-CONHNH$_2$/alginate microgel beads (100 μL aliquot of the original sample) after 20 mins at 90°C.

S4 Variable Temperature UV-Vis Studies

UV spectroscopy was employed to monitor the disruption of the DBS-CONHNH$_2$ network incorporated into the DBS-CONHNH$_2$/alginate microgel particles by applying a high temperature. The microgels used for this experiment were prepared as described above and dispersed in 1 mL of extra pure water. A 20 μL aliquot of the sample was diluted with extra pure water to a 2 mL total volume. The UV absorbance at 250 nm was firstly recorded at RT. The sample was then heated until a clear solution was obtained. The UV spectrum of the hot solution was then recorded, showing the characteristic DBS-CONHNH$_2$ peak. To ensure reproducibility, the data were collected in triplicate and the average is shown. To quantify the exact amount of DBS-CONHNH$_2$ released from the microgels after heating, a calibration curve was prepared using hot solutions of DBS-CONHNH$_2$ at different concentrations. The obtained results show that 0.0518 μmoles of DBS-CONHNH$_2$ are released from the microgels (20 μL aliquots of the original sample). It is therefore clear that self-assembled DBS-CONHNH$_2$ is present within the microgels and is subsequently released on heating, when disassembly can occur.

Considering that the initial microgel volume was 1 mL and assuming that heating completely destroys the DBS-CONHNH$_2$ network within the microgels, we calculated that the original microgel sample contained 0.0026 mmoles of DBS-CONHNH$_2$ (c.a. 1.23 mg), which corresponds to 41% of the DBS-C0NHNH$_2$ amount initially loaded. This data agrees with the NMR quantification study performed to evaluate the efficiency of the microgel fabrication method, which shows that a

considerable amount of DBS-CONHNH$_2$/alginate microgel particles is lost during the purification process.

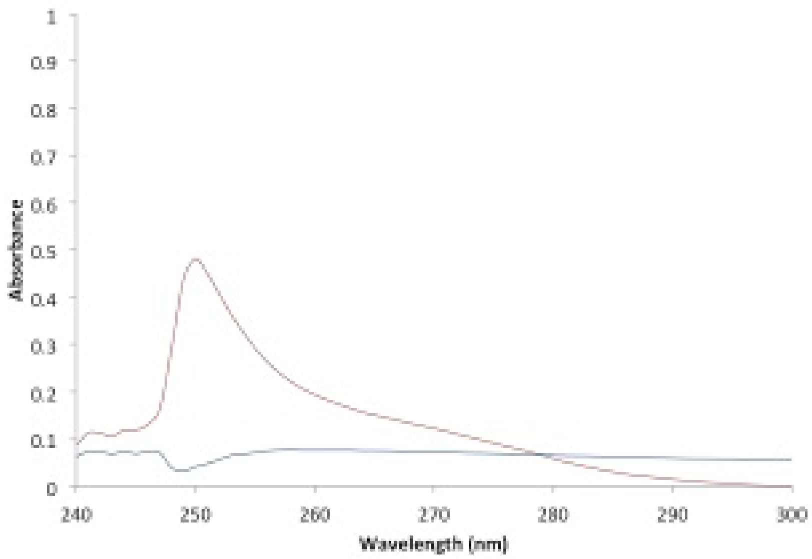

Figure S5. UV spectra of DBS-CONHNH$_2$/alginate microgels in water at RT (blue line) and after heating (red line).

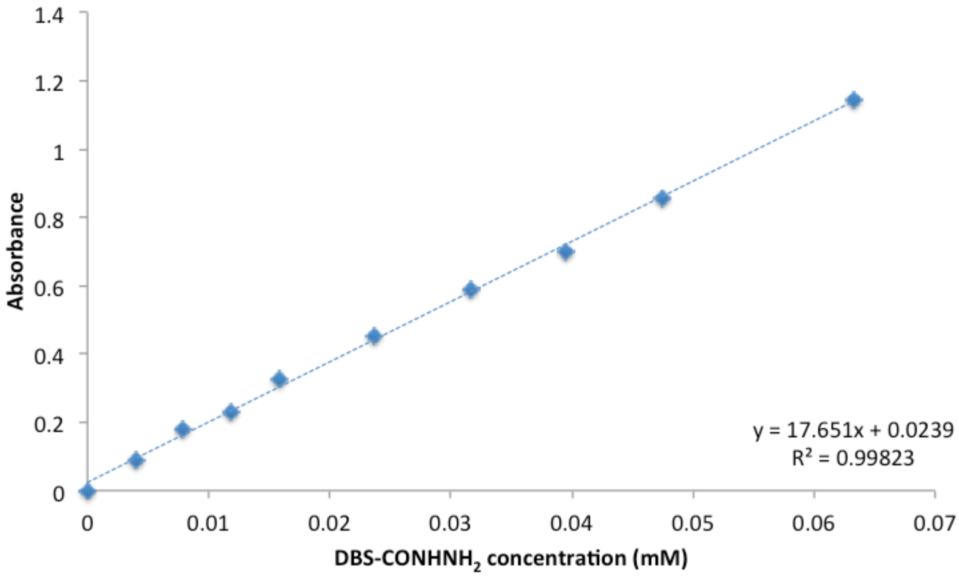

Figure S6. Calibration curve of DBS-CONHNH$_2$ hot solution at different concentrations (Abs recorded at 250 nm).

S5 Infrared (IR) spectroscopy

Xerogel samples for infrared were prepared by removing the solvent from the gels and microgels under high vacuum. A small amount of the resulting powder was placed into the infrared spectrophotometer and the spectra recorded in the range of 450-4000 cm^{-1}.

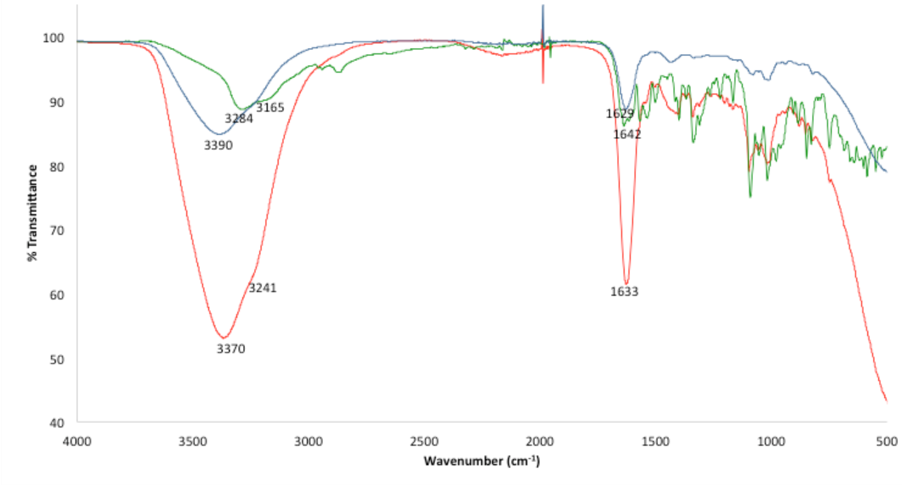

Figure S7. IR spectra of xerogels obtained from DBS-CONHNH$_2$ gel (0.4% wt/vol, green line), alginate gel beads (0.8% wt/vol, blue line) and DBS-CONHNH$_2$/alginate two-component gel beads containing 0.3% wt/vol of DBS-CONHNH$_2$ and 0.5% wt/vol alginate (red line).

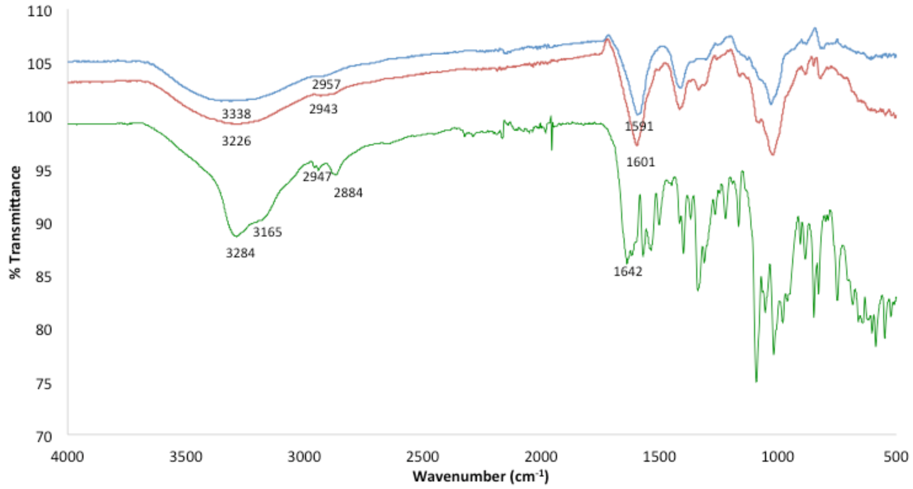

Figure S8. IR spectra of xerogels obtained from DBS-CONHNH$_2$ gel (0.4% wt/vol, green line), alginate microgels (0.8 % wt/vol, blue line) and DBS-CONHNH$_2$/alginate two-component microgels containing 0.3% wt/vol of DBS-CONHNH$_2$ and 0.5% wt/vol alginate (red line).

S6 Dynamic Light Scattering (DLS)

Samples for DLS experiments were prepared by diluting 100 μL of the microgels suspension with 1.4 mL of extra-pure water. All the DBS-CONHNH$_2$/alginate microgels samples were prepared using a 0.3% wt/vol concentration of the LMWG and a 0.5% wt/vol concentration of alginate, unless otherwise specified. The particle size distribution by intensity and by volume was recorded using a Malvern Zetasizer Nano.

Table S2. DLS data for DBS-CONHNH$_2$/alginate two-component hybrid microgels and alginate microgels prepared with different conditions.

Microgel	DBS-CONHNH$_2$ (wt/vol %)	Alginate (wt/vol %)	CaCl$_2$ (wt/vol %)	Oil : Water ratio	Span 80 (vol %)	Stirring speed (rpm)	Mean diameter (nm)	Std Deviation
Hybrid	0.3 %	0.5 %	5 %	40 : 2	2 %	1350	776.1	72.19
Hybrid	0.4 %	0.5 %	5 %	40 : 2	2 %	1350	754.9	61.8
Hybrid (after 30 days)	0.3 %	0.5 %	5 %	40 : 2	2 %	1350	820.3	49.7
Hybrid (after 10 months)	0.3 %	0.5 %	5 %	40 : 2	2 %	1350	908.1	81.31
Hybrid (in DMEM for 1 week)	0.3 %	0.5 %	5 %	40 : 2	2 %	1350	874.5	59.97
Hybrid (injected through a syringe)	0.3 %	0.5 %	5 %	40 : 2	2 %	1350	690.6	29.28
Hybrid	0.3 %	0.5 %	5 %	40 : 2	2 %	1000	1106.3	55.89
Hybrid	0.3 %	0.5 %	5 %	40 : 2	2 %	625	1054.3	151.6
Hybrid	0.3 %	0.75 %	5 %	40 : 2	2 %	1350	898.7	12.36
Hybrid	0.3 %	1.0 %	5 %	40 : 2	2 %	1350	959.5	24.32
Hybrid	0.3 %	0.5 %	2.5 %	40 : 2	2 %	1350	701.1 (pk 1), 55.21 (pk 2)	54.38 (pk 1), 16.25 (pk 2)
Hybrid	0.3 %	0.5 %	10 %	40 : 2	2 %	1350	776.2	52.29
Hybrid	0.3 %	0.5 %	5 %	40 : 2	1 %	1350	707.2	25.02
Hybrid	0.3 %	0.5 %	5 %	40 : 2	4 %	1350	1018	6.11
Hybrid	0.3 %	0.5 %	5 %	20 : 2	2 %	1350	962.5	122.2
Hybrid	0.3 %	0.5 %	5 %	60 : 2	2 %	1350	827.6	9.73
Alginate	-	0.8 %	5 %	40 : 2	2 %	1350	1178	25.58
Alginate (after 30 days)	-	0.8 %	5 %	40 : 2	2 %	1350	1174	55.89
Alginate	-	0	5 %	40 : 2	2 %	1000	1574.3	40.13
Alginate	-	0	5 %	40 : 2	2 %	625	1828	76.26

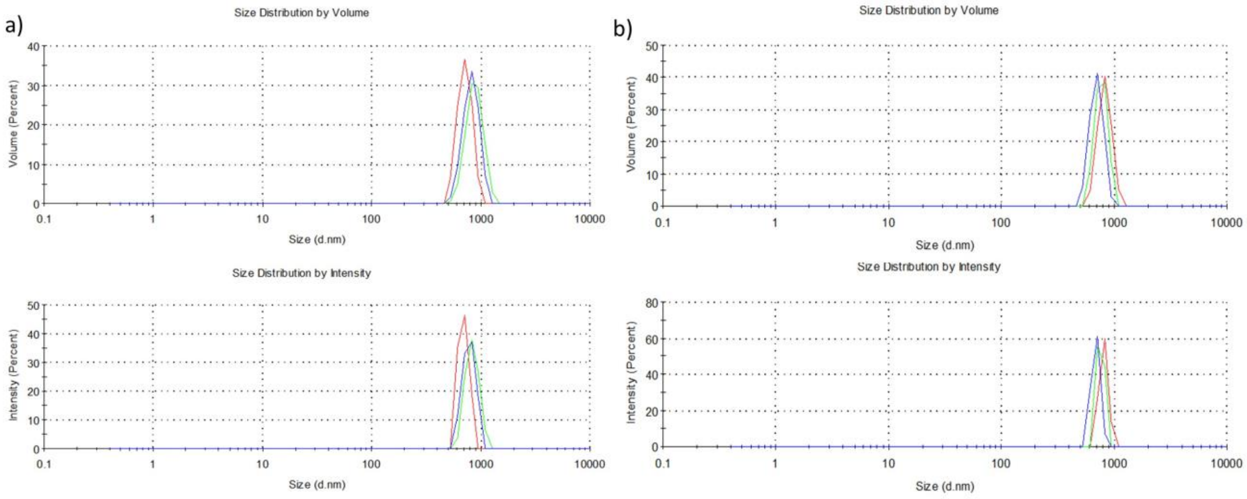

Figure S9. DLS data for DBS-CONHNH$_2$/alginate two-component microgels prepared by mixing at 1350 rpm, using a 0.3% wt/vol (a) or 0.4% wt/vol (b) concentration of DBS-CONHNH$_2$.

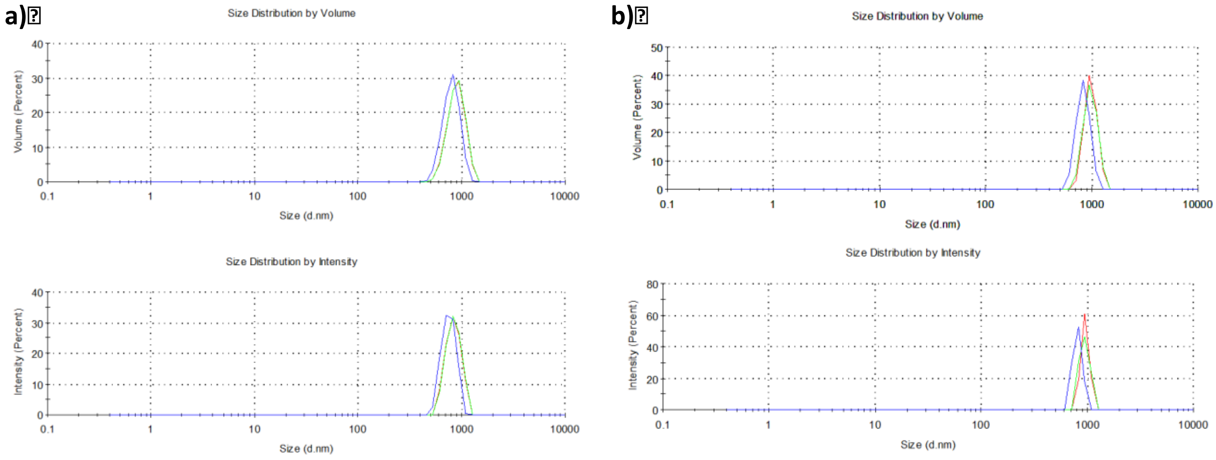

Figure S10. DLS data for DBS-CONHNH$_2$/alginate two-component microgels after 30 days (a) and 10 months (b). The samples were prepared by mixing at 1350 rpm speed.

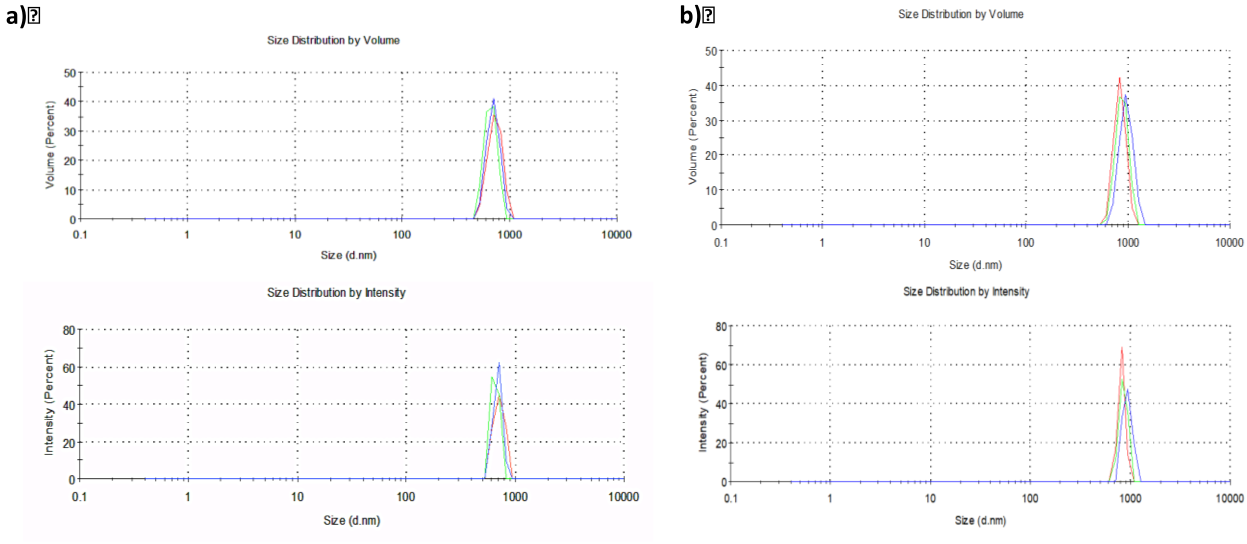

Figure S11. DLS data for DBS-CONHNH$_2$/alginate two-component microgels prepared with mixing at 1350 rpm. (a) Microgels had been injected through a syringe. (b) Microgels were dispersed in 10 mL Dulbecco's Modified Eagle's Medium (DMEM; FBS – 10%, P/S - 1%) and stored in the incubator at 37 °C for one week. After one week, the DMEM was removed by centrifugation and the sample was washed multiple times with extra pure water.

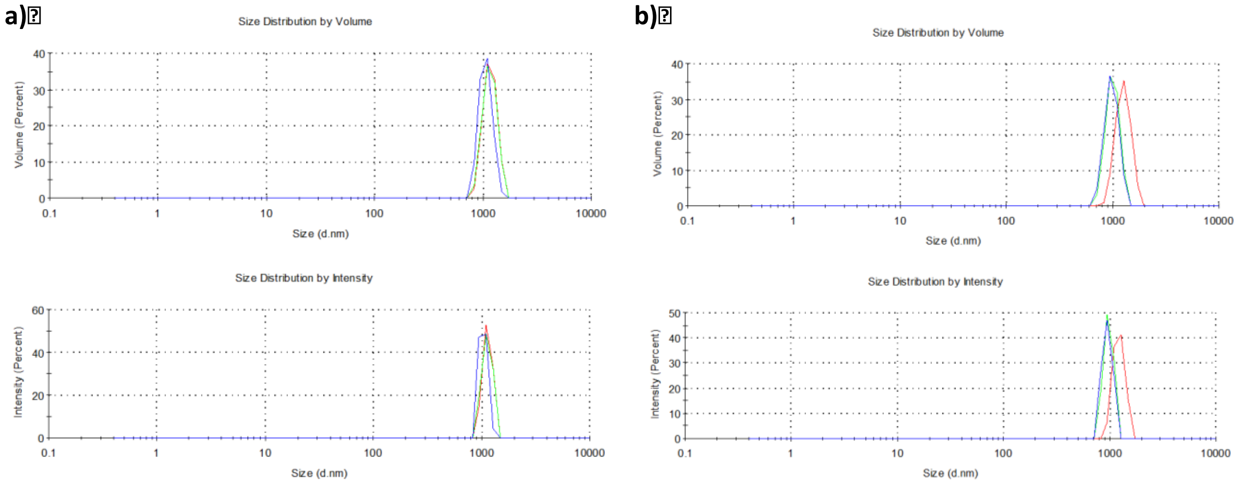

Figure S12. DLS data for DBS-CONHNH$_2$/alginate two-component microgels prepared by mixing at 1000 (a) and 650 rpm (b).

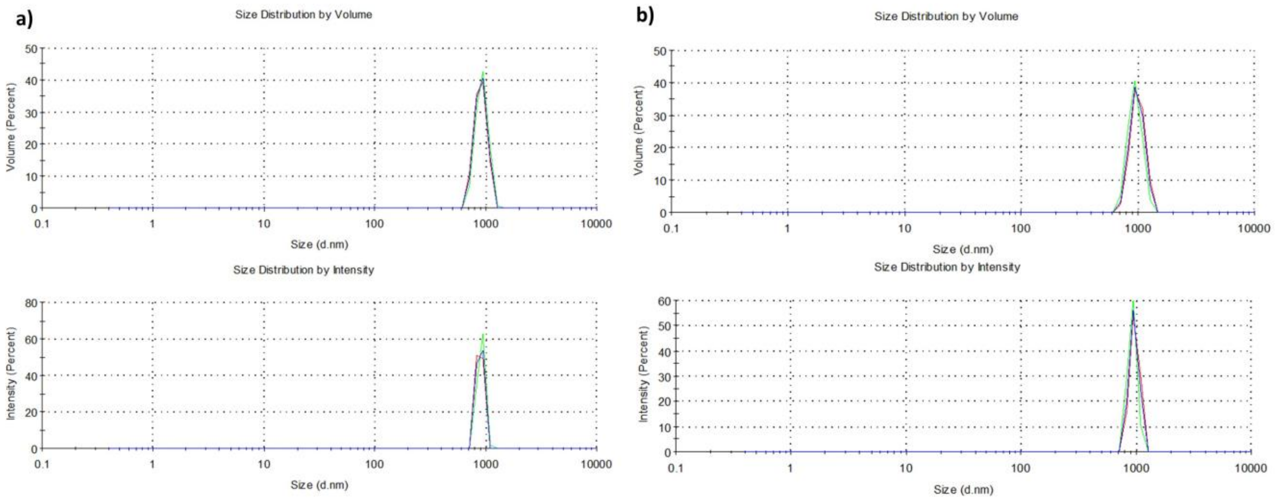

Figure S13. DLS data for DBS-CONHNH$_2$/alginate two-component microgels prepared by mixing at 1350 rpm, using a 0.75% concentration (a) or a 1.0% concentration (b) of alginate.

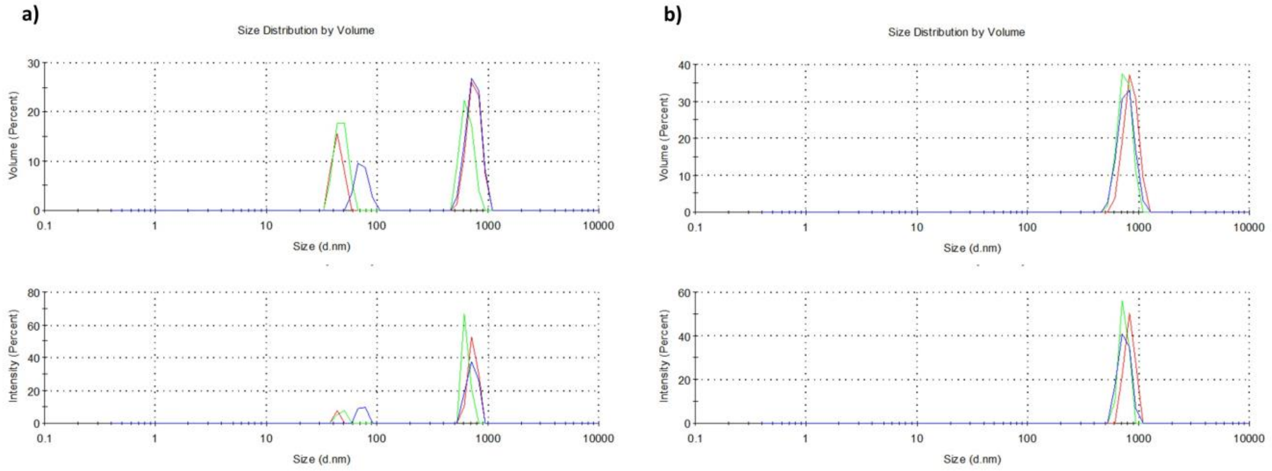

Figure S14. DLS data for DBS-CONHNH$_2$/alginate two-component microgels prepared by mixing at 1350 rpm, using a 2.5% concentration (a) or a 10% concentration (b) of $CaCl_2$.

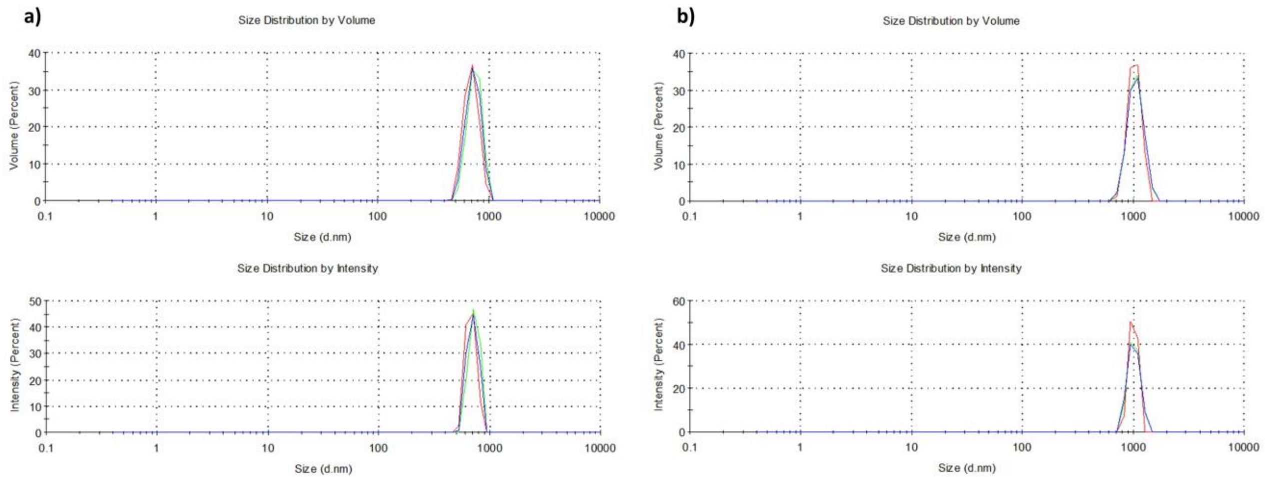

Figure S15. DLS data for DBS-CONHNH$_2$/alginate two-component microgels prepared by mixing at 1350 rpm, using a 1% concentration (a) or a 4% concentration (b) of Span 80.

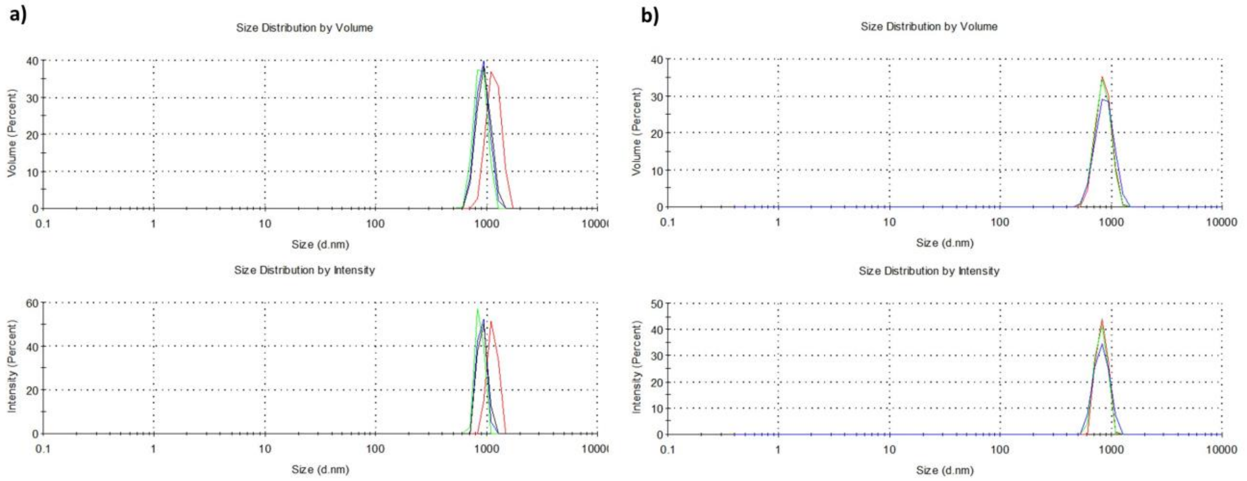

Figure S16. DLS data for DBS-CONHNH$_2$/alginate two-component microgels prepared by mixing at 1350 rpm, using a 20:2 (a) or a 60:2 (b) oil: water ratio.

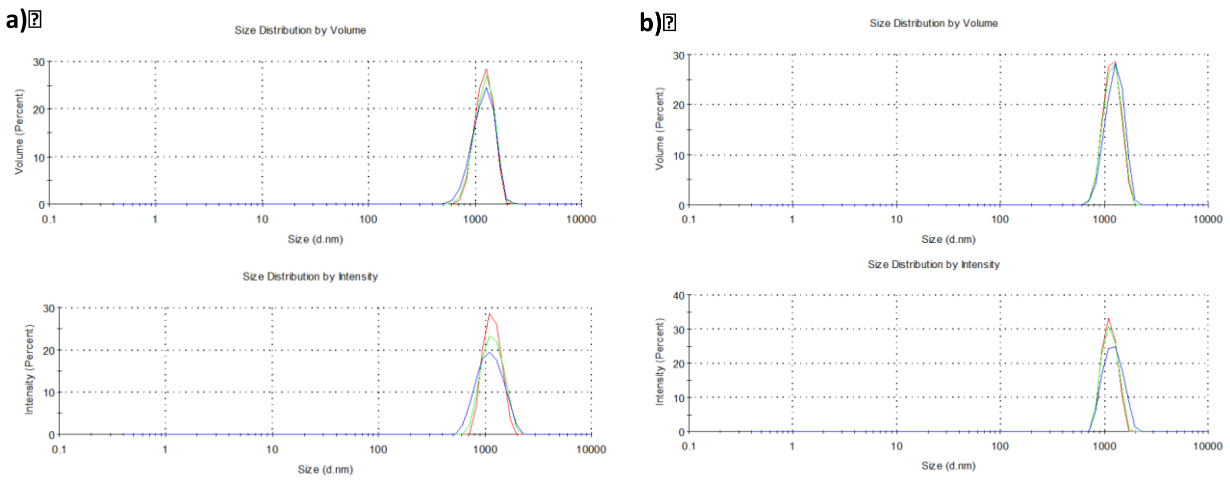

Figure S17. DLS data for alginate microgels immediately after preparation (a) and after 30 days (b). The samples were prepared by mixing at 1350 rpm speed.

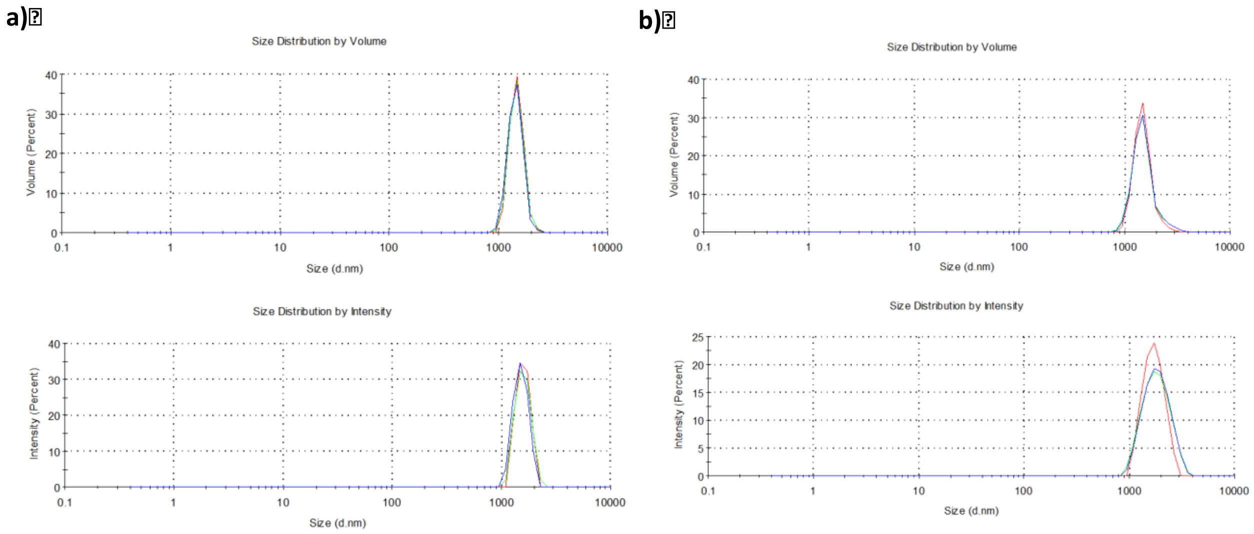

Figure S18. DLS data for alginate microgels prepared by mixing at 1000 (a) and 625 rpm (b).

S7 Optical Microscopy

Optical microscopy images were collected on a Zeiss stereo microscope. The gel beads were dehydrated through an ethanol series, then embedded in LR white resin. Sections were 1 μm thick. Once the section was dried on the slide, it was stained with Toluidine Blue (0.6% with 0.3% Na_2CO_3). The gel beads were prepared using 20 μL volumes using 0.3% wt/vol of DBS-$CONHNH_2$ and 0.5% wt/vol of alginate.

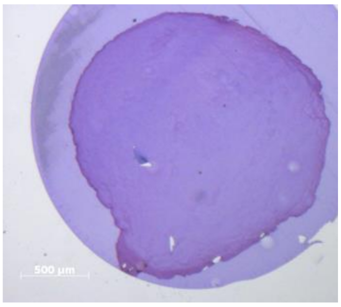

Figure S19. Optical microscopy image of DBS-CONHNH$_2$/alginate gel beads embedded in resin and stained with toluidine blue (scale bars 500 μm).

S8 Scanning Electron Microscopy (SEM)

S7.1 Preparation of samples for SEM. Gel beads samples for SEM were obtained by freeze drying the gels on copper shim pieces. The freeze-dried samples were then mounted on stubs and the images recorded. Microgels samples for SEM were dehydrated though an ethanol series whilst held in a millipore filter and subsequently treated with HMDS.

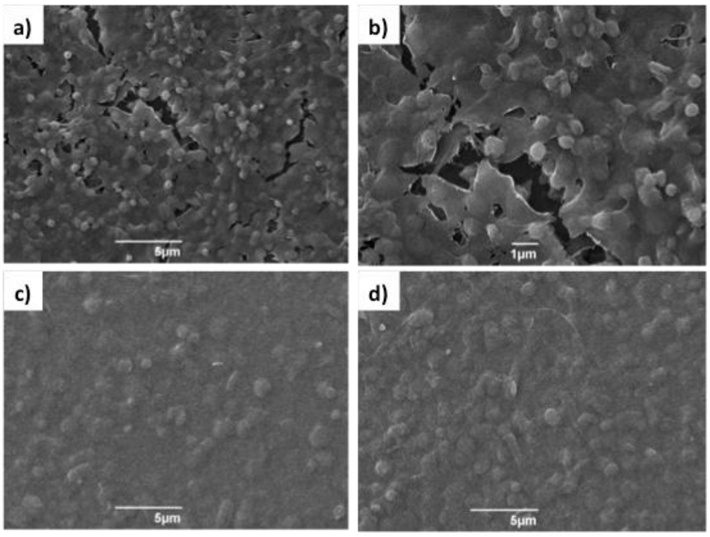

Figure S20. SEM images of DBS-CONHNH$_2$/alginate two-component microgels immediately after preparation (a, b) and after 30 days (c, d). Scale bars: 5 μm (a, c, d) and 1 μm (b).

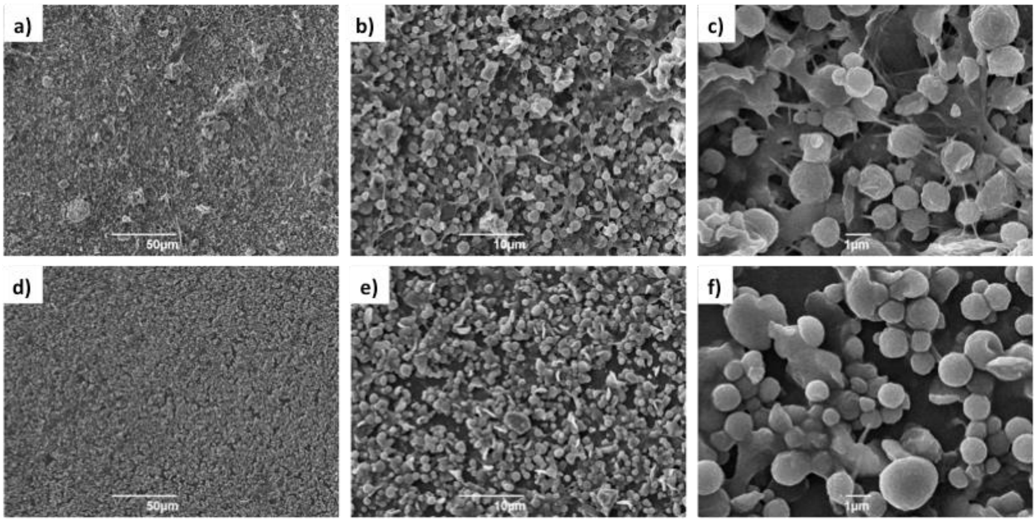

Figure S21. SEM images of alginate microgels immediately after preparation (a, b, c) and after 30 days (c, d, e). Scale bars: 50 (a, d), 10 (b, e) and 1 μm (c, f).

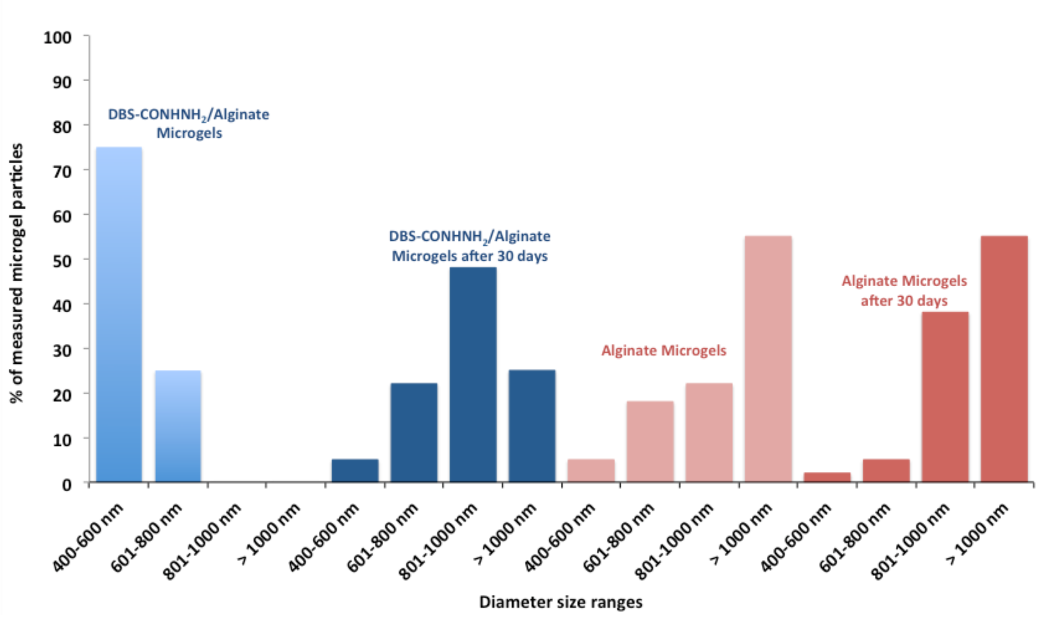

Figure S22. Diameter size distribution of microgel samples measured on 100 particles/sample on SEM images using the *ImageJ* Software.

S9 Drug release studies

S9.1 Heparin release assay. Gel samples for heparin release studies were prepared in 1 mL volume, as described in section S2. These were loaded with heparin by adding to each sample a 1 mM heparin solution (2 mL). To simplify sample manipulation for the microgels this procedure and subsequent steps were performed on the pellet formed immediately after the last centrifugation step of the preparation rather than on microgel suspensions. After 24 hours, the drug solution

was removed and used to quantify the exact amount of heparin incorporated into each gel by UV-vis spectroscopy. 2 mL of 10 mM Tris-HCl/150 mM NaCl buffer (pH 7.4) were placed on top of each gel and incubated at 37 °C for the duration of the study (24 hours). 65 μL aliquots of buffer were taken at regular time intervals. These aliquots were added to 1935 μL of MalB solution in 10 mM Tris-HCl/150 mM NaCl buffer (25.84 μM) and the UV-vis absorbance at 615 nm was monitored. To ensure reproducibility, the data were collected in triplicate and the average is shown. Control experiments with gels containing no heparin were also carried out. A calibration curve of heparin was obtained by adding 65 μL of known heparin concentrations in 10 mM Tris-HCl/150 mM NaCl buffer to 1935 μL of MalB solution in the same buffer and the absorbance spectra recorded.

S9.2 Heparin release data

Table S3. Percentage of heparin released over time from hydrogels. All the hybrid gels and microgels were prepared using 0.3% wt/vol DBS-CONHNH$_2$ and 0.5% wt/vol alginate.

Time (hours)	Heparin released from hydrogels (%)						
	DBS-CONHNH$_2$ (0.4 % wt/vol)	Alginate gel (0.8 % wt/vol)	Alginate gel beads (0.8 % wt/vol)	Alginate microgels (0.8 % wt/vol)	Hybrid gel	Hybrid gel beads	Hybrid microgels
0	0	0	0	0	0	0	0
0.5	11.98	10.14	3.66	14.83	12.76	11.15	16.49
1	21.52	19.23	9.14	24.47	16.41	19.89	25.03
2	30.50	34.88	28.47	33.96	27.43	36.38	32.00
3	37.40	44.78	37.84	41.08	33.03	49.15	39.93
4	40.76	47.045	36.73	41.04	33.93	49.89	42.31
5	41.51	46.94	38.09	40.61	33.50	50.80	41.91
6	41.48	48.80	39.13	39.65	34.93	49.42	39.82
7	46.39	47.46	38.24	37.97	34.25	50.37	40.70
8	43.90	47.66	40.16	-	36.79	47.03	-
24	46.17	50.58	42.56	38.50	35.30	51.49	36.90

S10 Biological studies

S10.1 Cell line (Y201 immortalized human mesenchymal stem cells – MSCs).[3] Y201 MSCs were grown in a T175 flask with Dulbecco's Modified Eagle's Medium (DMEM) with fetal bovin serum (FBS - 10%) and penicillin/streptomycin (P/S - 1%). To obtain the cells, the medium was removed from the flask and the cells washed with Dulbecco's phosphate buffer saline solution (11 mL).

Trypsin/EDTA (2 mL) was then added and the cells were incubated at 37°C for approximately five mins. When cell detachment was observed by optical microscopy, trypsin was neutralised with 9 mL DMEM (10% FBS, 1% P/S). The cells were then transferred in a tube and isolated by centrifugation. After centrifugation, the supernatant was removed and the cell pellet was dispersed in 5 mL DMEM (10% FBS, 1% P/S). Cell count was performed using a Countess Automated Cell Counter (Thermo Fisher) on a 10 μL aliquot of a stock solution obtained by mixing 20 μL of cell suspension with 20 μL of trypan blue.

S10.2 Gel preparation in transwell inserts and plate seeding. Gels were prepared as described in Section S2 in transwell inserts in 75 μL volume. Since the gel beads were prepared using 20 μL volume per bead, four gel beads were placed in each transwell insert. All the gels were sterilised through multiple washings with EtOH, then autoclaved water. Once ready, the gels were soaked with an aqueous heparin solution (0.2 or 0.4 mg in 200 μL) for 24 hours. The heparin incorporated into each sample was *c.a.* 50 % of the initial loading (*i.e.* 0.1 or 0.2 mg of heparin). After 24 hours, the supernatant was removed and the transwell inserts were then transferred to a 24 well plate in which the cells were seeded. The cells (25000/well) were seeded on the bottom of the wells in 24 well plates and covered with DMEM (10% FBS, 1% P/S - 500 μL).

S10.3 Microgel preparation in transwells and plate seeding. Microgel particles were prepared as described in Section S2 and sterilised through multiple washings with EtOH, autoclaved water and then cell culture medium. After the last washing, the supernatant was removed by centrifugation and a microgel pellet in DMEM was obtained. 75 μL of this pellet was transferred into each transwell insert. Each transwell insert was then immersed in a well of a 24 well plate containing heparin in cell culture medium (0.2 or 0.4 mg in 500 μL) for 24 hours. The heparin incorporated into each sample was *c.a.* 50 % of the initial loading (*i.e.* 0.1 or 0.2 mg of heparin). After 24 hours, the transwell inserts were transferred to a 24 well plate in which the cells were seeded. The cells (25000/well) were seeded on the bottom of the wells in 24 well plates and covered with DMEM (10% FBS, 1% P/S - 500 μL).

S10.4 Deep Blue viability assay. Cell viability was measured at day 0, 3 and 6 using the Deep Blue viability assay (BioLegend). 50 μL of Deep Blue was added to the culture medium into each well and the plates were incubated at 37°C for 2.5 hours. After this time, 10 μL aliquots were taken from each well and diluted with DMEM (190 μL) in a 96 well plate. Fluorescence was then

measured with a fluorimeter (excitation 530-570 nm and emission 600 nm). This experiment was performed in quadruplicates and average values are reported with the error bars representing standard error. 2 way ANOVA with Bonferroni post hoc testing after a D'Agostino & Pearson omnibus normality test was used to assess statistically significant differences in the four measures of viability between the cells that received heparin from the different types of gels and cells that were not exposed to heparin. Statistical significance is denoted by * = $p < 0.05$, ** = $p < 0.01$ and *** = $p < 0.001$.

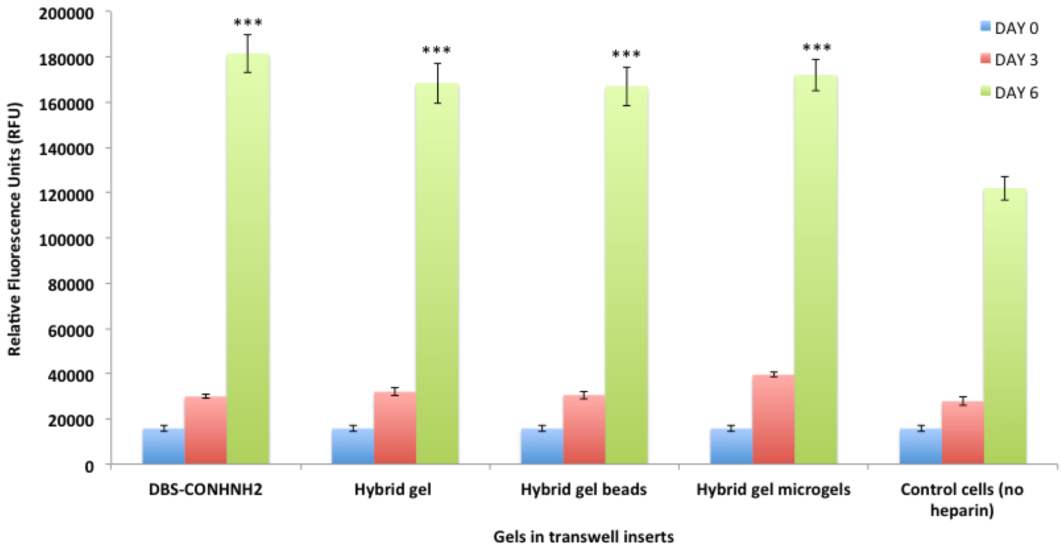

Figure S23. Deep Blue viability assay results at day 0, 3 and 6 for the different gels loaded with 0.2 mg of heparin. Statistical significance (comparing viability at day 6 of the cells that received heparin from the different types of gels and the cells that were not exposed to heparin) is denoted by *** = $p < 0.001$.

S9.5 Live cell staining – fluorescence microscopy. The plates were incubated with 13.3 μM calcein-acetoxymethyl solution (calcein-AM, 100 μL) in cell culture medium for 30 mins at 37°C. The staining solution was removed and the samples washed twice with DMEM (500 μL) for 15 mins. After this time, the cell culture medium was replaced with fresh DMEM (500 μL) and the images were collected using a fluorescence microscope.

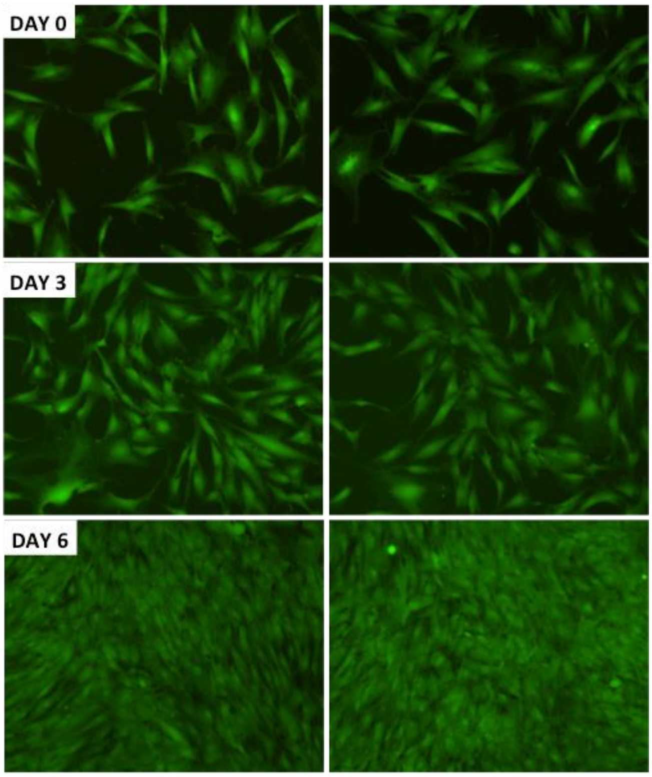

Figure S24. Fluorescence microscopy images of calcein AM stained control cells (not exposed to heparin) at day 0, 3 and 6.

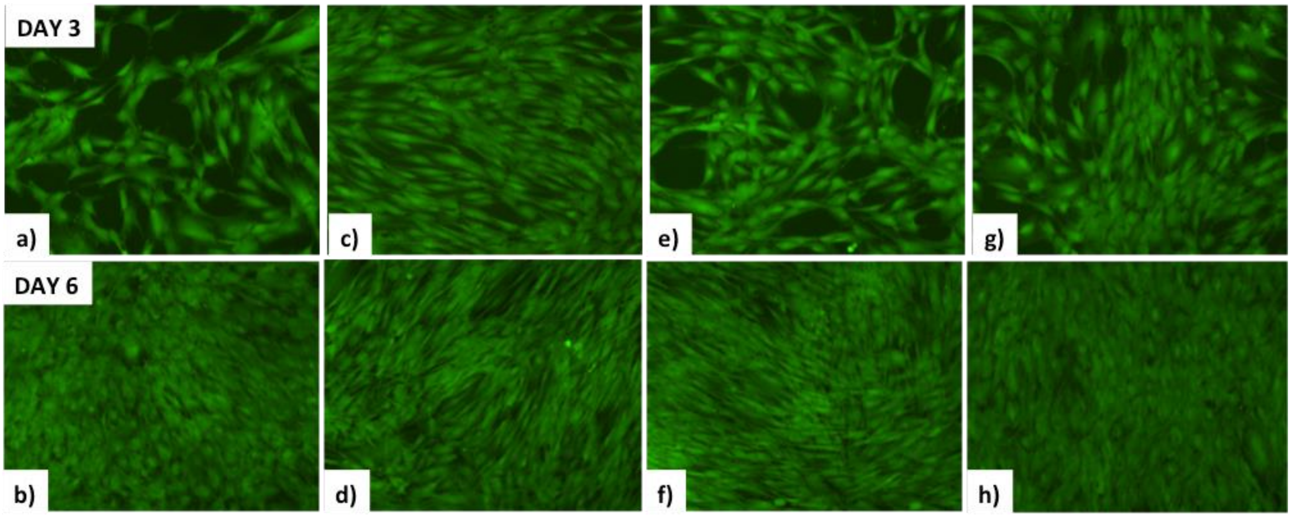

Figure S25. Fluorescence microscopy images at day 3 and 6 of calcein AM stained cells exposed to heparin (0.2 mg) released from DBS-$CONHNH_2$ gels (respectively a and b), DBS-$CONHNH_2$/alginate gels (respectively c and d), gel beads (respectively e and f) and microgels (respectively g and h).

S11 References

1. B. O. Okesola and D. K. Smith, *Chem. Commun.*, 2013, **49**, 11164-11166.
2. D. J. Cornwell, B. O. Okesola and D. K. Smith, *Soft Matter*, 2013, **9**, 8730-8736.
3. S. James, J. Fox, F. Afsari, J. Lee, S. Clough, C. Knight, J. Ashmore, P. Ashton, O. Preham, M. Hoogduijn, R. D. R. Ponzoni, Y. Hancock, M. Coles and P. Genever, *Stem Cell Reports*, 2015, **4**, 1004-1015.
